# Three dimensional multiphoton imaging of fresh and whole mount developing mouse mammary glands

**DOI:** 10.1186/1471-2407-13-373

**Published:** 2013-08-06

**Authors:** Michael D Johnson, Susette C Mueller

**Affiliations:** 1 Department of Oncology, Georgetown University School of Medicine, 3970 Reservoir Road NW, Washington, D.C. 20057-1469, USA

## Abstract

**Background:**

The applications of multiphoton microscopy for deep tissue imaging in basic and clinical research are ever increasing, supplementing confocal imaging of the surface layers of cells in tissue. However, imaging living tissue is made difficult by the light scattering properties of the tissue, and this is extraordinarily apparent in the mouse mammary gland which contains a stroma filled with fat cells surrounding the ductal epithelium. Whole mount mammary glands stained with Carmine Alum are easily archived for later reference and readily viewed using bright field microscopy to observe branching architecture of the ductal network. Here, we report on the advantages of multiphoton imaging of whole mount mammary glands. Chief among them is that optical sectioning of the terminal end bud (TEB) and ductal epithelium allows the appreciation of abnormalities in structure that are very difficult to ascertain using either bright field imaging of the stained gland or the conventional approach of hematoxylin and eosin staining of fixed and paraffin-embedded sections. A second advantage is the detail afforded by second harmonic generation (SHG) in which collagen fiber orientation and abundance can be observed.

**Methods:**

GFP-mouse mammary glands were imaged live or after whole mount preparation using a Zeiss LSM510/META/NLO multiphoton microscope with the purpose of obtaining high resolution images with 3D content, and evaluating any structural alterations induced by whole mount preparation. We describe a simple means for using a commercial confocal/ multiphoton microscope equipped with a Ti-Sapphire laser to simultaneously image Carmine Alum fluorescence and collagen fiber networks by SHG with laser excitation set to 860 nm. Identical terminal end buds (TEBs) were compared before and after fixation, staining, and whole mount preparation and structure of collagen networks and TEB morphologies were determined. Flexibility in excitation and emission filters was explored using the META detector for spectral emission scanning. Backward scattered or reflected SHG (SHG-B) was detected using a conventional confocal detector with maximum aperture and forward scattered or transmitted SHG (SHG-F) detected using a non-descanned detector.

**Results:**

We show here that the developing mammary gland is encased in a thin but dense layer of collagen fibers. Sparse collagen layers are also interspersed between stromal layers of fat cells surrounding TEBs. At the margins, TEBs approach the outer collagen layer but do not penetrate it. Abnormal mammary glands from an HAI-1 transgenic FVB mouse model were found to contain TEBs with abnormal pockets of cells forming extra lumens and zones of continuous lateral bud formation interspersed with sparse collagen fibers.

Parameters influencing live imaging and imaging of fixed unstained and Carmine Alum stained whole mounts were evaluated. Artifacts induced by light scattering of GFP and Carmine Alum signals from epithelial cells were identified in live tissue as primarily due to fat cells and in whole mount tissue as due to dense Carmine Alum staining of epithelium. Carmine Alum autofluorescence was detected at excitation wavelengths from 750 to 950 nm with a peak of emission at 623 nm (~602-656 nm). Images of Carmine Alum fluorescence differed dramatically at emission wavelengths of 565–615 nm versus 650–710 nm. In the latter, a mostly epithelial (nuclear) visualization of Carmine Alum predominates. Autofluorescence with a peak emission of 495 nm was derived from the fixed and processed tissue itself as it was present in the unstained whole mount. Contribution of autofluorescence to the image decreases with increasing laser excitation wavelengths. SHG-B versus SHG-F signals revealed collagen fibers and could be found within single fibers, or in different fibers within the same layer. These differences presumably reflected different states of collagen fiber maturation. Loss of SHG signals from layer to layer could be ascribed to artifacts rendered by light scattering from the dense TEB structures, and unless bandpass emissions were selected, contained unfiltered non-SHG fluorescence and autofluorescent emissions. Flexibility in imaging can be increased using spectral emission imaging to optimize emission bandwidths and to separate SHG-B, GFP, and Carmine Alum signals, although conventional filters were also useful.

**Conclusions:**

Collagen fibril arrangement and TEB structure is well preserved during the whole mount procedure and light scattering is reduced dramatically by extracting fat resulting in improved 3D structure, particularly for SHG signals originating from collagen. In addition to providing a bright signal, Carmine Alum stained whole mount slides can be imaged retrospectively such as performed for the HAI-1 mouse gland revealing new aspects of abnormal TEB morphology. These studies demonstrated the intimate contact, but relatively sparse abundance of collagen fibrils adjacent to normal and abnormal TEBS in the developing mammary gland and the ability to obtain these high resolution details subject to the discussed limitations. Our studies demonstrated that the TEB architecture is essentially unchanged after processing.

## Background

The utility of multiphoton and SHG imaging to probe the mammary gland structure and the implications of variations in collagen I fibrillar networks for mammary gland development have been recognized, and their use together with the use of transgenic models, biochemical, molecular genetics, and *in vitro* and *ex vivo* approaches have provided insight into the role of the extracellular matrix (ECM) in controlling normal mammary gland morphogenesis as well as tumorigenesis [1]. Multiphoton and SHG imaging provide multiple sources of information in unstained mammary gland tissues based on collagen fiber networks and FAD and NADH autofluorescence [2,3]. Recently, the implications of collagen fiber network structure for breast cancer prognosis have been explored and aligned collagen fibrillar structure defined as a prognostic signature for survival [4-8]. Biophysical studies of mammary gland remodeling and mechanosignaling and the intimate link of force production and response to collagen I network structures within the gland have been recently reviewed [9-16].

Clinical modalities of imaging tissues non-invasively have been applied to animal models to explore mammary gland structures [17,18]. These include the use of imaging live glands with reflectance confocal microscopy [17]. The advantage of these imaging approaches includes the ability to reconstruct 3D images of the glandular tissue and cross sectional imaging to elucidate the interior morphology of ductal tissue. Other live imaging modalities have been developed to non-invasively image tissue, and primary amongst them have been the use of fluorescence imaging to detect GFP expressed within the tissues of interest ([17,19] and references therein). More recently, these studies have been conducted using GFP-expressing mouse mammary glands that have been imaged together with ECM using SHG. In GFP-mice, SHG illustrates the association of extracellular matrix with the surface of tumors [2,20] and provides images of collagen fibrillar structure at high resolution [2].

Conditions for optimal imaging of collagen fibrillar structure using SHG-B have been reported by Zoumi et al. [21]. They found that at laser excitations less than 800 nm, signals from the ECM are a blend of SHG and multiphoton excitation signals from collagen, but at excitations greater than 800 nm signal is primarily from SHG [21]. Using a three dimensional (3D) organotypic tissue model, they demonstrate that the SHG-B intensity comprises a quadratic dependence upon excitation power, it decays exponentially with depth, and it is spectrally dependent [21].

The combination of SHG-B and immunocytochemistry has been used to demonstrate the association of collagen I fibers with terminal end buds in the developing mouse mammary gland and the promotion of collagen fibrillogenesis by macrophages [22]. These studies were conducted utilizing frozen sections of mammary gland as well as fixed propidium iodide-stained whole mount preparations. Interestingly, antibody staining of collagen I revealed no change in amount comparing mice homozygous for null mutation in CSF-1 with wild type, whereas SHG detection of collagen fibrils revealed a decrease in fibrillar structure in the CSF-1-deleted mouse mammary glands. These results were interpreted to mean that the anti-collagen I antibodies detected both fibrillar and less fibrillar forms of collagen [22]. In addition, SHG detected fibrils in places where antibody staining was negative. However, treatment of sections with collagenase confirmed that SHG and anti-collagen antibodies both recognized collagen I fibers [22].

In addition to the capacity to document collagen structure, SHG-B versus SHG-F potentially reveals information on the maturity of collagen fibers. Williams et al. have compared two day tendon with mature mouse tendon and conclude that fibrillar structure imaged in the SHG-F mode is more prominent compared with that imaged in the SHG-B, whereas in the mature tendon, the signals are identical [23]. They suggest that immature fibrils in SHG-B can be identified by punctuate structure compared with “segmental” collagen structure.

However, nearly all of these imaging techniques must be studied with live tissues in prospective studies and are limited by availability of animals and the proper timing required for lengthy imaging sessions. Various methods have been described for clearing fixed tissue for deep (millimeter) imaging in mouse organs such as brain tissue, and recently a method has been described that notably preserves fluorescence intensity [24,25]. However, the disadvantages include weeks of preparation time prior to imaging and tissue samples that cannot be archived. In mammary gland research where studies of large numbers of animals are required, typically mouse mammary glands are fixed, defatted, and prepared for permanent whole mounts stained with Carmine Alum. The archived tissue samples are later imaged using bright field microscopy. Alternative methods for whole mount preparations featuring different staining reagents and preparations have been reported that preserve antigenicity so that tissue can later be sectioned and immunostained [26].

Recently, we found that multiphoton microscopy can be used to retroactively image Carmine Alum-stained whole mounts to explore the morphology of hyperplastic glands and the inner structures of TEBs and ducts in three dimensions, specifically to determine whether ducts are filled with cells, retain a central hollow lumen, or abnormally contain multiple chambers or densely arranged lateral buds [27]. Imaging these prepared whole mounts allowed both retrospective examination of mammary gland samples and quantification of features using 3D imaging techniques as well as high resolution detection of collagen fiber and their association with terminal buds. Here we describe a method for multiphoton imaging combined with backward and forward scattered SHG signals to characterize mammary gland tissue and we also compare morphology of live GFP glands with Carmine Alum-stained as well as unstained whole mount preparations.

## Methods

### Materials

Wild-type FVB mice, or mice carrying an enhanced Green Fluorescent Protein (GFP) transgene under the control of the chicken beta-actin promoter coupled with the cytomegalovirus (CMV) immediate early enhancer (FVB.Cg-Tg (ACTBEGFP) B5Nagy/J strain were obtained from The Jackson Laboratories, Bar Harbor, Maine). HAI-1 transgenic FVB mice (unpublished), that overexpress the Kunitz-type protease inhibitor HAI-1 in their mammary glands were obtained from an in-house breeding colony. Mice were maintained and bred within the Division of Comparative Medicine animal facilities with unrestricted access to food and drinking water. Animals were examined at least once daily. All protocols involving the animals have been approved by the Georgetown University Animal Care and Use Committee (GUACUC) and carried out under the protocol No. 09043. After euthanizing animals according to the approved protocol, mammary tissue (the #3 or #4 glands) was harvested from 6–8 week or six month old wild-type, GFP, or HAI-1 mice (from 1–2 animals each) and spread out on microscope slides or cover glasses (Gold Seal #1) to mimic the arrangement of the tissue in the mouse as closely as possible. The glands were imaged immediately and then fixed and processed for whole mount preparation essentially as previously described [17]. Briefly, the whole mammary gland specimens were fixed in a 3:1 mixture of 100% ethanol and glacial acetic acid for 60 minutes at room temperature, washed with 70% ethanol for 15 minutes, and rinsed with deionized water. The glands were then stained overnight in Carmine Alum (1 g Carmine, #C1022, 2.5 g Aluminium potassium sulphate, #A7167, Sigma St Louis, MO in 500 ml of water) overnight at 4°C. The tissues were rinsed in deionized water and then dehydrated through graded alcohols and cleared in toluene prior to mounting with cover slips or microscope slides with Permount (Fisher Scientific, Pittsburgh PA).

### Microscopy

**Figure 1:**
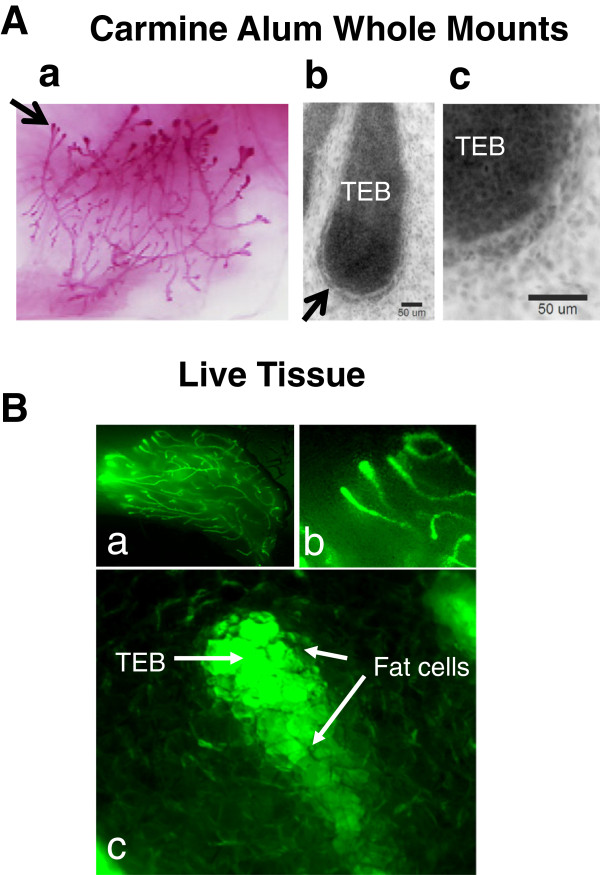
**Stereofluorescence microscopy of live mouse mammary gland, *ex vivo.*** GFP-mice were sacrificed and the 3rd inguinal gland was excised. **A a**-**c**. Carmine Alum-stained glands used for multiphoton imaging of normal TEBs in this study (see Figures 4, 8, and 11) were imaged using bright field optics, first with a Nikon SMZ1500 stereofluorescence **(A a)** and then using a Nikon E600 upright microscope (**A b**, Nikon 20X/ 0.5 N.A., and **c**, Nikon 40 X /0.95 N.A.). Scale bars = 50 μm. **B a**-**c**. Another mammary gland from a GFP-mouse was imaged using the stereofluorescence microscope. Fat cells, just visible in **B a**-**b** at lower magnifications, are viewed surrounding the ductal epithelium obscuring cellular details of the terminal end bud (TEB) in **B c**.

Confocal, bright field, and multiphoton images were collected using a Zeiss510/META/NLO microscope (Carl Zeiss, Thornwood, NY) with Zeiss EC Plan-Neofluar 10x/0.30 M27, a Zeiss LD LCI Plan Apo 25x/0.8 lens (working distance 0.57 at 0.17 mm cover glass), or a Zeiss c-Apochromat 40X/ 1.2 NA W corr. UV–VIS-IR M27. For transmitted SHG signals, a Zeiss achromatic condenser 0.8 H D Ph DIC was used with the polarizer replaced by an IR filter to block excitation IR in the emission. In Figure 1, images were made using a Nikon SMZ-1500 epi-stereofluorescence microscope (Melville, NY) equipped with a Pan Apo 1X Nikon lens and GFP filter or (Ex 480/40 nm, dichroic 505 nm, Em 535/50 nm) [17]. All confocal images were collected using a pinhole diameter of 1 Airy unit and multiphoton images were collected with the pinhole aperture set at 1000 μm (maximum) for internal detectors. Backward scattered second harmonic generation (SGH-B) was detected using an internal detector with maximal pinhole aperture 1000 μm) and forward scattered SHG (SHG-F) using a non-descanned detector (NDD) positioned in the transmission pathway, just above the condenser of the inverted microscope (ChD). Table 1 details imaging conditions for live and whole mount tissue experiments.

**Table 1:** Summary of imaging parameters (Zeiss LSM510/META/NLO specifications)

**Method**	**Laser**	**Emission**	**Imaging parameters**	**Image**
	**Excitation**			
Live Tissue	860 nm	390-465 nm	Ch2 BP 390–465 IR	Figure 2
	(5.8%)	500-550 nm	Ch3 BP 500–550 IR	
			MBS: HFT KP650	
			DBS1: Mirror	
			DBS2: NFT 490	
			FW1: None	
Live Tissue	860 nm	390-465 nm	ChS BP 393-436	Figure 3A-C
	(5.8%)		ChD	
			MBS: HFT KP650	
			DBS1: Mirror	
			DBS2: NFT 490	
			FW1: None	
	860 nm	390-465 nm	ChS BP 393-436	Figure 3D
	(5.8%)	500-550 nm	Ch2 BP 500-550	
			ChD	
			MBS: HFT KP650	
			DBS1: Mirror	
			DBS2: NFT 490	
			FW1: None	
Live Tissue	860 nm	390-465 nm	Ch2 BP 390–465 IR	Figure 4 (Live Tissue)
	(5.8%)	500-550 nm	Ch3 BP 500–550 IR	
		ChD	ChD (unfiltered)	
			MBS: HFT KP650	
			DBS1: Mirror	
			DBS2: NFT 490	
			FW1: None	
Carmine Alum WM	860 nm	390-465 nm	Ch2 BP 390–465 IR	Figure 4 (Whole Mount)
	(5.8%)	500-550 nm	Ch3 BP 500–550 IR	
		ChD	ChD (unfiltered)	
			MBS: HFT KP650	
			DBS1: Mirror	
			DBS2: NFT 490	
			FW1: None	
Carmine Alum WM	543 nm (50.5%)	565-615 nm	Ch3 BP 565–615 IR	Figure 5A,B
			ChD	
			MBS: HFT 488/543/633	
			DBS1: NFT 635 VIS	
			DBS2: NFT 545	
			FW1: None	
Carmine Alum WM	750 nm (2.1%)	565-615 nm	Ch3 BP 565–615 IR	Figure 5C
			MBS: HFT KP650	
			DBS1: NFT KP545	
			DBS2: NFT 545	
			FW1: None	
			BF NDD	
Carmine Alum WM	750 nm (5.8%)	565-615 nm	Ch3: BP 565–615 IR	Figure 6
			MBS: HFT KP650	
			DBS1: Mirror	
			DBS2: NFT 545	
			FW1: None	
Carmine Alum WM	860 nm (5.8%)	390-465 nm	Ch2 BP 390–465 IR	Figure 7
		650-710 nm	Ch3 BP 650–710 IR	
		ChD	ChD (unfiltered)	
			MBS: HFT KP650	
			DBS1: Mirror	
			DBS2: NFT 490	
			FW1: None	
Carmine Alum WM	860 nm (5.8%)	393-436	ChS BP 393-436	Figure 7B
		ChD	ChD (unfiltered)	SHG-B/SHG-F image only
			MBS: HFT KP650	
			DBS1: Mirror	
			DBS2: NFT 490	
			FW1: None	
Carmine Alum WM	860 nm (5.8%)	390-465 nm	Ch2 BP 390–465 IR	Figure 8
		650-710 nm	Ch3 BP 650–710 IR	
		ChD	ChD (unfiltered)	
			MBS: HFT KP650	
			DBS1: Mirror	
			DBS2: NFT 490	
			FW1: None	
Carmine Alum WM	860 nm (5.8%)	390-465 nm	Ch2 BP 390–465 IR	Figure 9
		650-710 nm	Ch3 BP 650–710 IR	
		ChD	ChD (unfiltered)	
			MBS: HFT KP650	
			DBS1: Mirror	
			DBS2: NFT 490	
			FW1: None	
Carmine Alum WM	750, 800, 830, 860, 890, 950 nm (0.1%)	Lambda scan	ChS:361-704	Figure 10
		361-704 nm	MBS: HFT KP650	
			DBS1: None	
			DBS2: None	
			FW1: None	
Carmine Alum WM	860 nm (5.8%)	390-465 nm	Ch2 BP 390–465 IR	Figure 11 (Ex 860/ Em 650–710)
		650-710 nm	Ch3 BP 650–710 IR	
		ChD	ChD (unfiltered)	
			MBS: HFT KP650	
			DBS1: Mirror	
			DBS2: NFT 490	
			FW1: None	
	750 nm (5.8%)	565-615 nm	Ch3: BP 565–615 IR	Figure 11 (Ex 750/ Em 565–615)
			MBS: HFT KP650	
			DBS1: Mirror	
			DBS2: NFT 545	
			FW1: None	
Unstained WM	735, 860, 960 nm (0.1%)	Lambda scan	ChS:361-704	Figure 12A, B
		361-704 nm	MBS: HFT KP650	
			DBS1: None	
			DBS2: None	
			FW1: None	
	860 nm (5.8%)	390-465 nm	Ch2 BP 390–465 IR	Figure 12C-E
		500-530 nm	Ch3 BP 500–530 IR	
		or	or	
		650-710 nm ChD	Ch3 BP 650–710 IR ChD (unfiltered)	
			MBS: HFT KP650	
			DBS1: Mirror	
			DBS2: NFT 490	
			FW1: None	
Unstained WM	860, 890 nm (5.8%)	390-465 nm	Ch2 BP 390–465 IR	Additional file 13: Figure S11
		500-530 nm	Ch3 BP 500–530 IR	
		ChD	ChD (unfiltered)	
			MBS: HFT KP650	
			DBS1: Mirror	
			DBS2: NFT 490	
			FW1: None	
	800, 890 nm (0.1%)	Lambda scan	ChS:361-704	Additional file 13: Figure S11
		361-704 nm	MBS: HFT KP650	
			DBS1: None	
			DBS2: None	
			FW1: None	
Unstained WM	800, 890 nm (0.1%)	390-465 nm	Ch2 BP 390–465 IR	Additional file 13: Figure S11
		500-530 nm	Ch3 BP 500–530 IR	
		ChD	ChD (unfiltered)	
			MBS: HFT KP650	
			DBS1: Mirror	
			DBS2: NFT 490	
			FW1: None	
Carmine Alum WM	860 nm (5.8%)	390-465 nm	Ch2 BP 390–465 IR	Additional file 1: Figure S1
		650-710 nm	Ch3 BP 650–710 IR	
		ChD	ChD (unfiltered)	
			MBS: HFT KP650	
			DBS1: Mirror	
			DBS2: NFT 490	
			FW1: None	
Live Tissue	860 nm (0.1%)	Lambda scan	ChS:361-704	Additional file 2: Figure S2
		361-704 nm	MBS: HFT KP650	
			DBS1: None	
			DBS2: None	
			FW1: None	
Carmine Alum WM	860 nm (5.8%)	390-465 nm	Ch2 BP 390–465 IR	Additional file 4: Figure S3
		650-710 nm	Ch3 BP 650–710 IR	
		ChD	ChD (unfiltered)	
			MBS: HFT KP650	
			DBS1: Mirror	
			DBS2: NFT 490	
			FW1: None	
Carmine Alum WM	860, nm (5.8%)	390-465 nm	Ch2 BP 390–465 IR	Additional file 5: Figure S4
		650-710 nm	Ch3 BP 650–710 IR	
		ChD	ChD (unfiltered)	
			MBS: HFT KP650	
			DBS1: Mirror	
			DBS2: NFT 490	
			FW1: None	
Carmine Alum WM	860 nm	Lambda scan	ChS:361-704	Additional file 7: Figure S5A,B
	(0.1%)	361-704 nm	MBS: HFT KP650	Additional file 11: Figure S9B
			DBS1: None	
			DBS2: None	
			FW1: None	
Carmine Alum WM	860 nm (5.8%)	390-465 nm	Ch2 BP 390–465 IR	Additional file 7: Figure S5C
		650-710 nm	Ch3 BP 650–710 IR	
		or	or	
		565-615	Ch3 BP 565–615 IR	
		ChD	ChD (unfiltered)	
			MBS: HFT KP650	
			DBS1: Mirror	
			DBS2: NFT 490	
			FW1: None	
Carmine Alum WM	860 nm (5.8%)	390-465 nm	Ch2 BP 390–465 IR	Additional file 8: Figure S6
		650-710 nm	Ch3 BP 650–710 IR	
		or	or	
		565-615	Ch3 BP 565–615 IR	
		ChD	ChD (unfiltered)	
			MBS: HFT KP650	
			DBS1: Mirror	
			DBS2: NFT 490	
			FW1: None	
Carmine Alum WM	860 nm (5.8%)	390-465 nm	Ch2 BP 390–465 IR	Additional file 9: Figure S7
		650-710 nm	Ch3 BP 650–710 IR	
		or	or	
		565-615	Ch3 BP 565–615 IR	
		ChD	ChD (unfiltered)	
			MBS: HFT KP650	
			DBS1: Mirror	
			DBS2: NFT 490	
			FW1: None	
Unstained WM	860 nm	Lambda scan	ChS:361-704	Additional file 10: Figure S8
	(0.1%)	361-704 nm	MBS: HFT KP650	
			DBS1: None	
			DBS2: None	
			FW1: None	
Unstained WM	860 nm (0.1%)	390-465 nm	Ch2 BP 390–465 IR	Additional file 11: Figure S9A, C
		500-530 nm	Ch3 BP 500–530 IR	
		ChD	ChD (unfiltered)	
			MBS: HFT KP650	
			DBS1: Mirror	
			DBS2: NFT 490	
			FW1: None	
Unstained WM	860 nm (0.1%)	390-465 nm	Ch2 BP 390–465 IR	Additional file 12: Figure S10
		500-530 nm	Ch3 BP 500–530 IR	
		ChD	ChD (unfiltered)	
			MBS: HFT KP650	
			DBS1: Mirror	
			DBS2: NFT 490	
			FW1: None	

Filters and Dichroics (LSM 510 Manual).

***KP*= Low Pass Filter**. Example: KP 685 passes wavelengths less than 685 nm to the detector.

***LP*= High Pass Filter**. Example: LP 505 passes wavelengths higher than 505 nm to the detector.

***BP*= Bandpass Filter.** Example: BP 565–615 passes wavelengths 565 nm to 615 nm to the detector.

***HFT*= Main Dichroic Beam Splitter**. Example: HFT 488/543 Excitation wavelengths (488 and 543 nm) sent to the sample.

Passes all other wavelengths up to the detection channels. Example: HFT KP 700/488 Works as both a dichroic and a low pass filter. Will pass all wavelengths less than 700 nm, except for 488 nm, which hits sample, but is not sent to detector.

***NFT*= Secondary Dichroic Beam Splitter.** Example: NFT 543 Wavelengths above 543 nm, pass straight through. Wavelengths less than this amount are reflected 90 degrees.

***NT*= Neutral Density Filter.** Example: Works as an attenuation filter. NT 80/20 passes 80% of the light, cuts 20% of light.

An NDD located in the transmitted light path just beyond a Zeiss ACHR COND 0.8 HD PH DIC condenser and IR filter is a useful addition to image transmitted SHG and/or transmitted fluorescence. The major signal from the ChD transmittance NDD detector is the SHG-F originating from collagen fibers and Carmine Alum or GFP signals which are much weaker by comparison. For these studies, these signals were not separated, although addition of emission filters could be used to isolate the transmitted SHG signal if desired. Additional file 1: Figure S1 illustrates the Zeiss510/META/NLO AIM software specific setup to obtain SHG-B, Carmine Alum, and SHG-F signals using a single excitation of 860 nm and internal filter set combinations in Ch2, Ch3, and ChD.

### Image processing and analysis

All images are presented in raw form (no post processing) with the exception of the Z-image stacks which were processed for 3D reconstruction as described using Image J 1.44p (NIH, Bethesda, MD), Metamorph Offline ver. 7.7.1.0 (Molecular Devices, Sunnyvale, CA), or Zeiss 510 META (with Physiology Software ver. 3.5 and Multiple Time Series) Software ver. 3.5 (Carl Zeiss MicroImaging, LLC, Thornwood, NJ). Contrast and brightness settings were changed identically for comparisons.

## Results

### Live imaging of GFP-mouse mammary gland *ex vivo*

Excised mammary glands from GFP-mice were first examined using conventional bright field with a stereofluorescence microscope (Figure 1A a-c). Next, by way of comparison, live, excised glands from GFP-mice were examined with fluorescence imaging using the same stereofluorescence microscope (Figure 1B a-c). Notably, at higher magnification, the terminal end buds (TEBs) were obscured by the presence of surrounding fat cells in the stroma (Figure 1B c).

Multiphoton imaging of mammary glands was performed to improve resolution and to achieve the benefits of three dimensional imaging (3D). First, emission from GFP and SHG signals were studied in live mouse mammary glands (Additional file 2: Figure S2A) and skin tissue (Additional file 2: Figure S2B-C) by collecting emission scans from 361–704 nm. At excitation 860 nm, GFP emission appeared as a peak at 506 nm with a shoulder at approximately 549 nm (Additional file 2: Figure S2A-B, green curve, eGFP peak). SHG-B appeared as a sharp peak centered at 431 nm (Additional file 2: Figure S2B, SHG-B peak). Images were extracted from the lambda data at Em 404–446 nm, 446–478 nm, 500–532 nm, and 596–730 nm (Additional file 2: Figure S2C). The SHG-B signal was reasonably well separated (Em 404–446, Additional file 2: Figure S2C). However, background contributed to the GFP image at Em 500–532 nm. Images lacking the GFP signal and containing only background signal were observed at Em 446–478 nm and Em 596–703 nm (Additional file 2: Figure S2C). Notably, the peak of the autofluorescent signal was Em 495 nm, whereas the peak of GFP was Em 506 nm.

**Figure 2:**
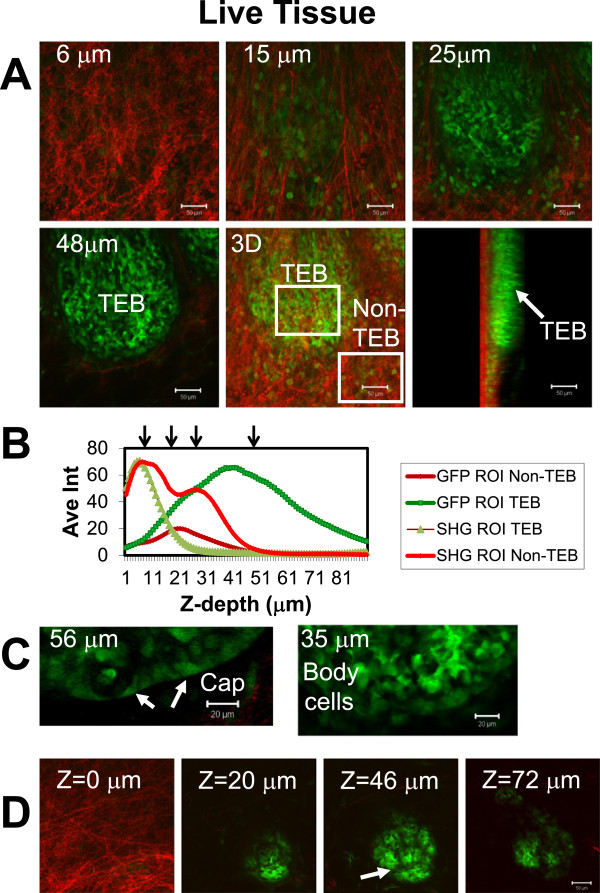
**Multiphoton microscopy of live GFP-mouse mammary gland, *ex vivo.*** In each series **(A**-**C** and **D)**, imaging acquisition began at the margin of the mammary gland resting on the coverslip and extended to depths of 90 μm **(A)** and 72 μm **(D)** including a terminal end bud (TEB). The margin of the mammary gland is identified by the dense collagen/ ECM layer visualized by SHG-B (red) that is absent in the gland interior. The TEB volume (GFP images, green) excludes the SHG-B signal. **(B)** is average intensity plotted on the Y-axis and Z-image depth plotted on the X-axis. Arrows indicate image depths shown in **A** (6 μm, 15 μm, 25 μm, and 48 μm). The boxed regions of the 3D reconstruction image (**A**, 3D) includes the position of R01’s used for quantification of SHG-B and GFP signal intensities in **B** (ROIs are indicated by white boxes “TEB” and “non-TEB”). In **C**, the same TEB was imaged with a Zeiss c-Apochromat 40X/ 1.2 NA W corr. lens. Although this lens has a shorter working distance, cellular details can be obtained at higher resolution when the TEB is relatively close to the surface of the mammary gland. At Z = 35 μm, the body cells of a TEB (“Body cells”) appear very bright and separated by non-GFP containing spaces. At Z = 56 μm, a position midway into the TEB in the Z-dimension, cap cells at the tip of the TEB are seen as a continuous layer with an outer, smooth margin (arrows, “Cap”). **D**. A dense array of cells (GFP) is poorly imaged since the adipose tissue scatters light and obscures GFP in a pattern reflecting the shape of the fat cells (arrow, z = 46 μm). Scale bars **A, D** = 50 μm; in **C**, 20 μm.

Using bandpass filters and single track imaging with MP excitation at 860 nm, SHG-B and GFP signals were collected for a single living, *ex vivo* TEB to generate Z-stacks (Figure 2; Table 1 for imaging details). In red, the SHG-B images depict the changing arrangement of collagen fibers with depth, i.e. a more linear and parallel arrangement of fibers at z =15 μm compared with the more disordered and wavy appearance at a shallower depth of imaging at z = 6 μm (Figure 2A). SHG-B signal disappeared deeper into the tissue beyond the TEB (Figure 2A-B, D). In addition to TEB epithelial cells, stromal cells were observed scattered within the ECM layer surrounding the TEB (Figure 2A, z = 25 μm). The TEB epithelial cells seen at higher magnification include TEB body cells and cap cells (Figure 2C: z = 56 μm, Cap, arrows; z = 35 μm, Body cells). However, TEBs imaged beneath the SHG-B-positive fiber layer lying within the fat tissue of the mammary gland appeared to have shadow artifacts arising from the outline of the fat cells (Figure 2D, arrow).

**Figure 3:**
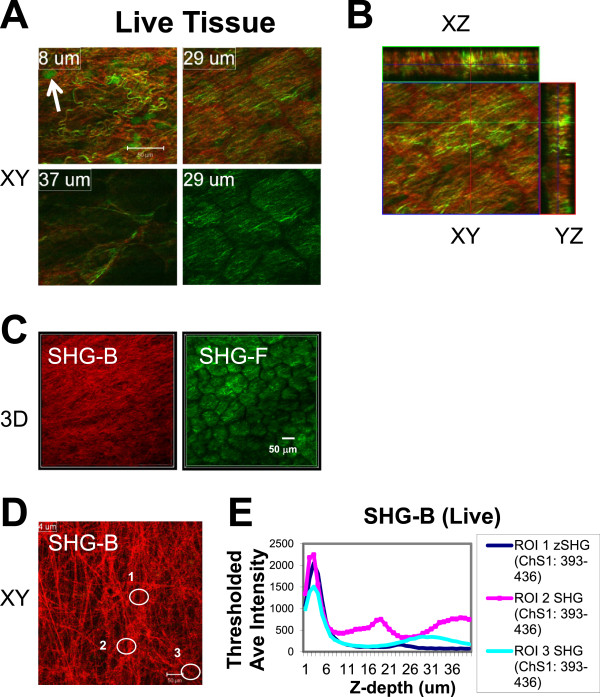
**Comparison of reflected (SHG-B) and transmitted SHG-F signals reporting surface collagen layers.** At Ex 860 nm, SHG-B (ChS: 393–436 nm; red) and SHG-F (ChD, green) images were collected in a Z-series beginning at the lateral margin of the gland. **A**-**B**. The patterns of SHG-B and SHG-F are not identical since single color fibers appear red (SHG-B) or green (SHG-F) as shown in XY planes **(A)** and in orthogonal images (XY; XZ; YZ) **(B)**. **C**. 3D images were prepared using Metamorph Offline Ver. 7.7.7.1 (“Open in 4D viewer”). The surface shown rested adjacent to the coverslip surface. The SHG-F view bears the pattern of the outlines of fat cells, whereas the view of fibers provided by SHG-B is not affected. **D**-**E**. A single XY slice reveals details of the fiber orientation and was taken at a depth of 4 μm. The average intensities of SHG-B signals for three different ROIs were calculated and plotted against Z-depth on the X-axis. The thickness of the fibrillar layer is on the order of 6 μm, although SHG is detected variably deeper into the tissue. Scale bars = 50 μm.

We next compared SHG-B and SHG-F signals obtained from live tissue. Interestingly, the two signals, SHG-B and SHG-F, imaged different fibrils, even when collected near the surface of the outer ECM layer, whether or not they had the same orientations (Figure 3A and B, compare green and red fibrils in 8 μm and 29 μm XY planes). SHG-F was unfiltered and included the entire spectrum available in ChD, thus contributions from GFP were included so that some cell bodies were observed in Figure 3A (8 μm, arrow). Whereas SHG-B signal was relatively unaffected by the light scattering due to the outlines of fat cells, SHG-F was dramatically affected as visualized in a 3D reconstruction (Figure 3C). This is likely due to the fact that the transmitted signal SHG-F passes through the entire thickness of the tissue containing fat cells prior to detection of the SHG-F by the non-descanned detector (NDD) which was located in the light path after the condenser. In single XY slices, fiber orientation was easily observed by SHG-B (Figure 3D). Average intensity scans at three different ROIs reveal the variation in depth and/or intensity of the SHG-B signal (Figure 3E, graph, peaks of intensity).

### Comparison of live and whole mount tissue imaging of mammary glands

**Figure 4:**
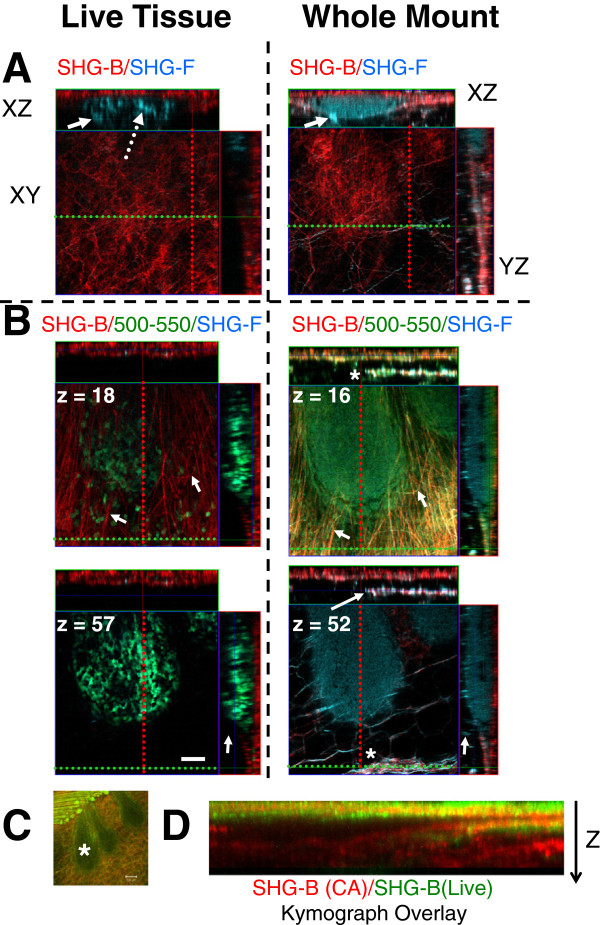
**Live GFP-mouse mammary gland compared with the identical whole mounted gland.** GFP-mouse mammary gland from a 4.5 week old mouse (gland number 3) was prepared as described in *Methods* and imaged using a Zeiss LD PAPO 25x/0.8 lens (Table 1). **A**. SHG-B/SHG-F were imaged in **A** and SHG-B/ BP 500-550/SHG-F were imaged in **B**. Orthogonal views are compared. In the live tissue XZ view (on top left), blue represents the unfiltered ChD transmitted signal which includes SHG-F (white arrow) and the GFP fluorescence (blue of epithelial cells, dotted arrow). In whole mount tissue (at right), the SHG-F (blue, white arrow) is more intense. The signal from unfiltered ChD also includes the Carmine Alum fluorescence (remaining blue associated with epithelial cells). The XY view illustrates SHG-B (red) of the surface fibrillar layer (Z = 1 and Z = 6, respectively, for live and whole mount). **B**. Orthogonal views made at different Z-depths illustrate increased information provided by combination of SHG signals (SHG-B, red and ChD unfiltered, including both SHG-F plus GFP in the live and SHG plus Carmine Alum fluorescence in the whole mount). Arrows indicate points of comparison between live and whole mount tissue. Asterisks indicate a fiber-associated vessel. **C**. The TEB shown in **A**-**B** is included in this lower magnification image taken using a Zeiss Neofluar 10x/0.30 lens (asterisk, green GFP, red, SHG-B). **D**. A kymograph was generated from a line with average 100 pixel width. The line was similarly placed on the image of the live and whole mount TEB and the image merged. SHG-B from the whole mount appears in red, and the SHG-B from the live appears in green. **A**-**B**, Scale bar = 50 μm, **C**, Scale bar = 100 μm.

A comparison of the cellular and fibrillar structure of TEBs and the surrounding stroma was performed in live and whole mount tissue to determine whether major artifacts are introduced by whole mount preparation. Thus, the same TEBs were imaged in living tissue immediately after excising the gland and then again following fixation, defatting, and Carmine Alum staining. The living and whole mount tissue was first compared using SHG-B and SHG-F imaging of collagen fibers together with either GFP or Carmine Alum fluorescence (Figure 4, Live (GFP) Tissue and Whole Mount, solid arrows indicate the same fibers in live versus whole mount images). The XY scan of the surface fibrillar layer revealed that the fibers were compressed around the TEB in the whole mount preparation, whereas in the live tissue they were less compressed (Figure 4A).

The SHG-F signal was barely detectable in the live tissue, but was prominent in the whole mount (Figure 4A, Live Tissue and Whole Mount, blue, solid arrows). The GFP in live cells is included in the SHG-F (unfiltered) channel (Figure 4A, Live Tissue, dashed arrow, blue). Similarly, in the whole mount, both the SHG and the Carmine Alum signal are seen in the unfiltered SHG-F channel (Figure 4A, Whole Mount, blue, arrow indicates SHG-F, remaining blue is Carmine Alum). In live tissue, individual epithelial cells in the TEB as well as surrounding stromal cells are visualized (Figure 4B, Live Tissue, z = 18). Surrounding the TEB, a layer of fibers is best observed in the Whole Mount preparation (Figure 4, Whole Mount, XZ views). In addition, in the whole mount, SHG-B and SHG-F signals were acquired significantly deeper into the tissue; in fact a layer of fibrils associated with a blood vessel is apparent in the whole mount that is not imaged in the live tissue (Figure 4B, Whole Mount, asterisks XY view, arrows in XZ and YZ views). Figure 4C is a low magnification view of the TEB imaged in Figure 4A-B (asterisk). A kymograph was generated for live and whole mount tissues along a line at approximately the same site and an overlay created to further illustrate the difference in imaging depth of SHG-B between the live and whole mount tissue (Figure 4D, red is whole mount SHG-B and green is live SHG-B, asterisk indicates TEB position). Thus, the combination of SHG-B and SHG-F, together with Carmine Alum signals in whole mounts increases the depth at which tissue architecture can be observed compared with live mammary gland tissue.

### Structures revealed by multiphoton imaging of Carmine Alum fluorescence in mammary gland whole mounts

**Figure 5:**
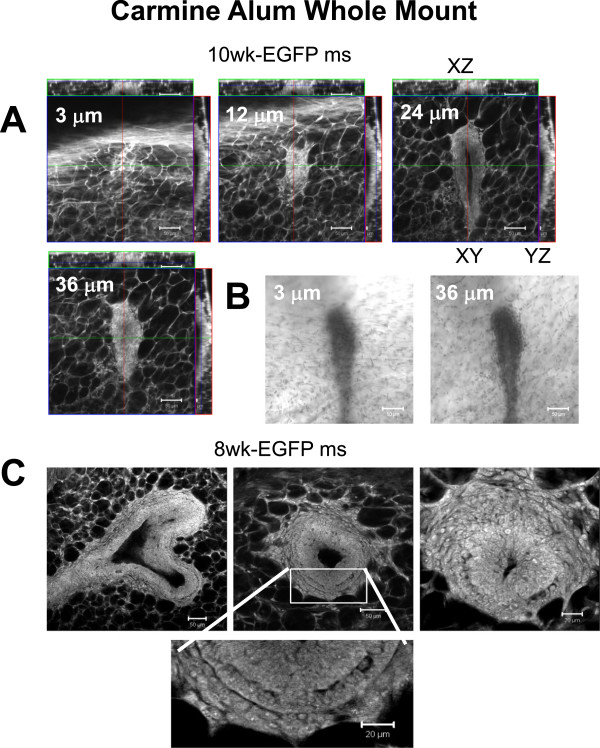
**Confocal and multiphoton imaging of Carmine Alum fluorescence in fixed mammary gland whole mounts.** Confocal and bright field imaging of TEBs close to the surface of a mammary gland reveals the morphology and arrangement of TEBs relative to the plane of the coverslip. **A**. Imaging of a 10 week mammary gland from an EGFP mouse was performed using a 543 nm HeNe laser and conventional confocal imaging of single XY planes since the TEB was close to the surface of the gland. A typical filter set for imaging of red dyes was used (emission bandpass of 565–615 nm). **B**. Bright field imaging of the same TEB was not very informative. **C**. Multiphoton imaging of an EGFP-mouse mammary gland was performed using a Chameleon XR Ti-Sapphire laser tuned to 750 nm (2.1%), and PMT Ch3 was used with bandpass filter 565–615 and pinhole set open to 1000 μm. Scale bars = 50 μm except for the inset indicated by a white box, 20 μm.

Most experiments with mouse glands involve large complex sample collections in which multiple glands (minimum of 2 usually) in multiple animals are harvested for each treatment or time point. Carmine Alum stained whole mount preparations are typically prepared from these experiments so that the data can be analysed according to researcher and instrument availability without concern for sample deterioration. Previously, we had determined that Carmine Alum is fluorescent and can be imaged in 3D [27]. For those experiments, excitation of the Ti-Sapphire MP laser was set to 750 nm and emission was collected in the red channel, 565–615 nm, although mammary gland whole mounts can also be imaged using confocal microscopy with a visible HeNe green laser with excitation at 543 nm (compare Figure 5A. Ex 543 nm with Figure 5C, Ex 750 nm). Bright field imaging of the Carmine Alum staining at the same magnification was not illuminating compared with the fluorescence confocal imaging (compare Figure 5B to 5A).

**Figure 6:**
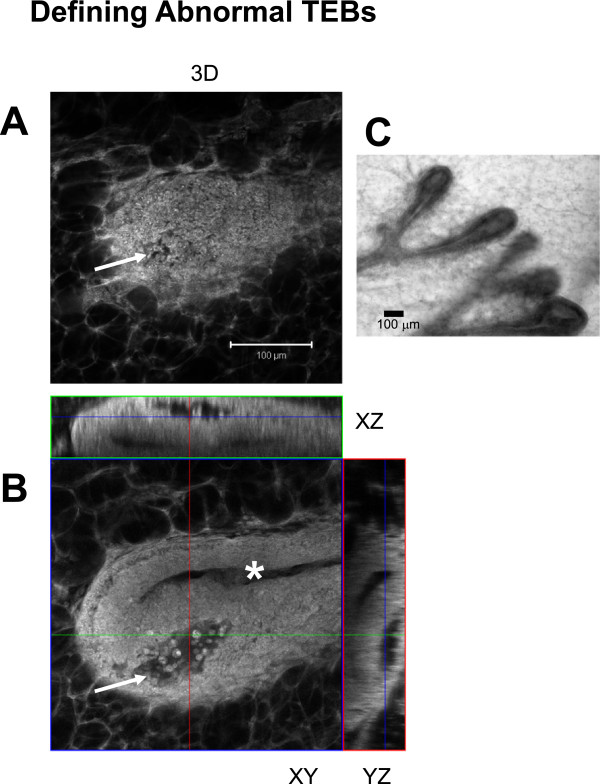
**Detection of abnormal TEB lumen.** A Carmine Alum stained TEB from a HAI-1 mouse was sampled using Z-slice spacing of 1 μm for 74 μm. **A**. A 3D reconstruction was made using Zeiss AIM software. The arrow points to a defect on the surface of the TEB which was stained with Carmine Alum. Imaging was performed using Ex 750 nm and Em bandpass 565–615 nm. **B**. An individual Z-slice at z = 24 μm and orthogonal XZ and YZ slices illustrate the central lumen of the duct (asterisk) and the abnormal pocket of cells forming an extra lumen near the tip of the TEB (arrow). **C**. A conventional bright field image from an HAI mouse taken using a Nikon E600 upright microscope equipped with a 10X/ 0.3 N.A. Nikon lens. Scale bars = 100 μm.

As an example of the power of 3D imaging of Carmine Alum staining, mammary glands from HAI-1 mice were imaged in the same manner as Figure 5C (Ex 750/EM 565–615 nm). A 3D reconstruction of a TEB is shown in Figure 6A and orthogonal views in Figure 6B. These mice exhibit delayed mammary gland development, and analysis of H&E stained paraffin sections of the mammary glands (but not bright field images) had suggested that the structure of the TEBs was abnormal. 3D imaging of the TEBs made it much easier to appreciate and quantify the nature of their abnormal structure. Furthermore, the evidence for these abnormalities could not be obtained by examination of bright field images (Figure 6C). Orthogonal views (XZ and YZ) to a single plane XY image reveal that the central lumen is not connected with a pocket forming a defect reaching to the surface of the TEB (Figure 6A-B, arrows for abnormal pocket, asterisk marks the central ductal lumen). Examination of a movie through the Z-planes confirms a lack of connection between the two lumens (Additional file 3: Movie S1). Observations of dozens of TEBs to determine the number of abnormal pockets per TEB would have been extremely time consuming and would have to be done using serial sections of traditional paraffin embedded tissue.

**Figure 7:**
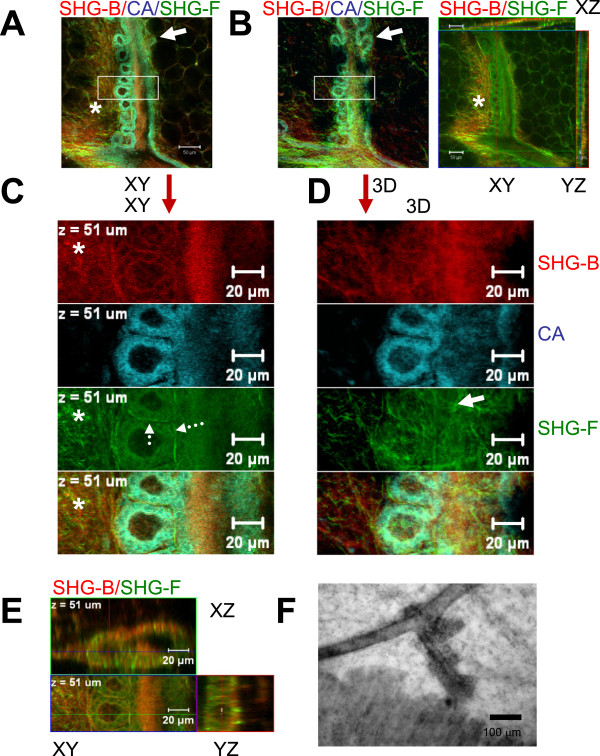
**Imaging abnormal features of developing mammary glands using optimal SHG-B/Carmine Alum/SHG-F approach. A**. A three color, single slice (XY) from a Z-stack of images of 46 μm thickness is presented from a Carmine Alum whole mount (HAI-1 mouse gland). **B**. The three-color, 3D projection is shown (3D) as well as orthogonal views which were acquired separately for just the SHG-B and SHG-F signals using a narrower bandwidth in the ChS (META) detector for SHG-B. Arrows in the lower magnification upper panel indicate differences between the XY and 3D views of the lateral branches, which otherwise look similar indicating that they are mainly viewed in a single XY orientation relative to the stage. **C**. A higher magnification view of the boxed inset region in **A** (XY) reveals details of Carmine Alum staining and SHG-B and SHG-F. The fibers visualized using SHG are concentrated near the duct and lateral buds (asterisks in **A**, **B** and **C**). The dotted arrows shown in C indicate fibers (SHG-F) between and around the lateral buds. **D**. A higher magnification view of the boxed inset region in B reveals the 3D view of Carmine Alum staining with SHG-B and SHG-F. **E**. Orthogonal views including XY, XZ, and YZ reveal two lateral buds viewed as two color image of SHG-B and SHG-F (E). **F**. A conventional bright field image of a similar duct taken using a Nikon upright microscope equipped with a 10X/ 0.3 N. **A**. Nikon lens. Scale bars = 50 μm in **A** (top panel) and 20 μm elsewhere except **F**, 100 μm.

In a final experiment, details of the ductal side branches were explored in a mouse model of HAI-1 in which potential abnormalities had been identified previously at the bright field level (Johnson, unpublished data). Imaging of SHG-B, Carmine Alum and SHG-F was performed and the resulting XY, 3D and orthogonal XY, XZ, and YZ images were compared (Figure 7A-D). It is apparent that the single XY slice and 3D views reveal multiple details not available from paraffin sections or bright field imaging of Carmine Alum-stained whole mounts (Figure 7F). First, the many lateral buds present along the duct appear mostly in a single plane (XY) (Figures 7A and B, compare XY slice and 3D views). Second, the lower power views (Figure 7A and B) and the inset of each shown in Figure 7C and D) reveal the association of SHG-B and SHG-F positive fibrils with the duct. The increased fibrillar texture is observed adjacent to the duct on the side containing the lateral buds (Figure 7C, XY views, asterisks). The 3D views illustrate that the fibrillar layer indeed surrounds both the duct and the lateral buds (Figures 7D, 3D, solid arrow), and extends between the buds (Figure 7C, dashed arrows). Carmine Alum staining serves to intensify the epithelial signal, but visualization of ductal cells is still possible when the Carmine Alum image plane is deleted in the multicolour view since unfiltered fluorescence from Carmine Alum appears in the transmitted channel in this particular experiment (Figure 7E).

**Figure 8:**
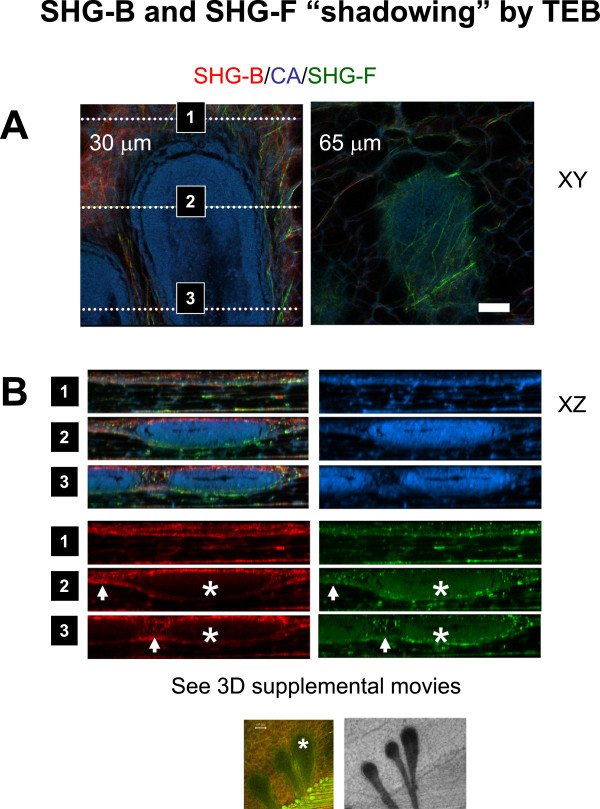
**TEBs are associated with linear configurations of collagen fibers.** Using a GFP-mouse mammary gland from a 4.5 week old mouse (gland number 3), a TEB near the surface of the mammary gland was imaged to a depth of 98 μm using SHG-B and SHG-F together with Carmine Alum fluorescence. **A**, Linear arrays of collagen fibers were observed around and between TEBs (Z = 30, and Z = 65 μm). **B**. XZ line scan presentation of the results illustrates the shadowing of the SHG signals by the TEB (asterisks). 3D movies of the Z-stack from this and an adjacent view can be found in the Additional file (Additional file 6: Movies S4-5). Scale bar = 50 μm.

Figure 8 illustrates more completely the architecture of the collagen fibers surrounding a normal TEB in a Carmine Alum stained whole mount and that the SHG-B signals often predominate at the surface of the gland closest to the cover glass compared with SHG-F signals that are also apparent beneath the TEB (further into the tissue away from the cover glass) (Figure 8A-B). The SGH-B signal (red), although intense on the surface of the gland, is diminished beneath the TEB (Figure 8B, red, note position of TEBs marked by asterisks), whereas the SHG-F signal (green) appears most intensely on the deeper edge of the TEB (Figure 8B, green, note position of TEBs marked by asterisks). In XY regions imaged on either side of the TEB, both SHG-B and SHG-F signals nearer and farther from the cover glass are collected in the final image (Figure 8B, arrows) illustrating the likelihood of a “shadowing” effect on the SHG signals by the Carmine Alum-stained TEB. In the case of SHG-B, the shadowing effect occurs on the far side of the TEB deeper into the tissue, whereas the shadowing effect for SHG-F occurs on the near side of the TEB. This is due to the differing light paths to the detector for each. However, we infer that SHG-B signal is indeed present on the lower aspect of the TEB since in adjacent areas it is present where TEB tissue is not interposed between the source of the SHG-B and the epi-detector. Similarly, we infer that the SHG-F signal is present above or on the near side of the TEB since in areas adjacent to the TEB it appears (Figure 8B, arrows). We conclude that the combination of SHG-B and SHG-F gives a good idea where layers of fibers occur, but in a non-quantitative manner.

**Figure 9:**
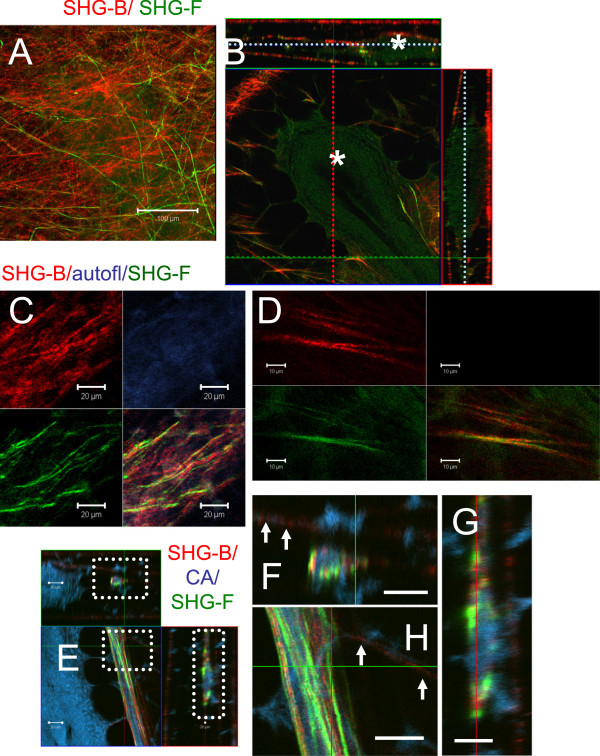
**SHG-B and SHG-F identify different collagen fibers.** A Z-stack of images 78 μm deep was collected of a TEB from a Carmine Alum whole mount. **A**. A two-color image of SHG-B and SHG-F signals illustrates that even in one XY plane, two types of collagen fibers are identified. **B**. An orthogonal view of the same region showing SHG-B and SHG-F signals at various depths. Three layers of fibers are seen with the TEB which is sandwiched between two of the layers, asterisk. Dashed lines indicate planes of associated images; red for YZ, green for XZ, and blue for XY. **C** and **D**. SHG-B (red) / 500–550 nm (autofluorescence, blue)/ SHG-F (green) images reveal that SHG-B and SHG-F signals are not identical despite their arrangement in parallel arrays. In **D**, Individual fibers can be seen to have both SHG-B and SHG-F signals. Typically the signal from SHG-B (red) has more texture, whereas the signal from SHG-F (green) is smoother and finer in appearance. **A**-**B**, **E**-**G**. Small vessels contain associated fibers with both SHG-F and SHG-B showing strong signal intensity compared with with TEB-associated fibers that are indicated by arrows. F-G are insets from E indicated in E by dashed boxes. **E** scale bar = 50 μm; **C**, **F**-**H**, scale bar = 20 μm; **D**, scale bar = 10 μm.

The difference in SHG-B and SHG-F signals cannot be explained solely on light scattering properties of Carmine Alum-stained epithelial cells within the TEBs. Both SHG-B and SHG-F signals are seen in fibers within the same layer and in fibers associated in parallel orientations adjacent to TEBs (Figure 9A-D) as well as associated with blood vessels (Figure 9E-H). Some fibers appear to emit both SHG-B and SHG-F signals (Figure 9D). Fibers associated with blood vessels create more intense SHG signals compared with fibers in the rest of the mammary gland stroma, but similarly contain both SHG-B and SHG-F signals originating from different fibers (Figure 9E-H, arrows point to non-vessel associated fibers).

Additional file 4: Figure S3 illustrates the loss of SHG-F signal when a TEB is positioned between the collagen fibrils and the NDD. In this example, the SHG-B signal from collagen fibers surrounding the blood vessels is nearer to the coverslip than the TEB and so the reflected signal is efficiently recovered by the internal PMT detector (Additional file 4: Figure S3A). In contrast, the SHG-F signal must first travel though the thickness of the stromal tissue and then is scattered by the TEB and fails to be detected by the NDD (Additional file 4: Figure S3A). Although the laser penetrates to the TEB, the farther half of the Carmine Alum-stained TEB (relative to the coverslip) is often poorly imaged, but some detail is imaged in the in the SHG-F channel due to the autofluorescent or Carmine Alum bleed through (Additional file 4: Figure S3A-B, note blue to green transition of the TEB with Z-depth). There is a clear loss of SHG-B signal when the Carmine Alum-stained TEB is situated between the coverslip and the source of the SHG-B signal (Additional file 4: Figure S3C, note SHG-B signal in red channel). Thus, using both the signals from SHG-B and SHG-F, the approximate architecture of collagen fiber layers can be discerned. To illustrate this, three dimensional views of a TEB associated with layers of fibers imaged by SHG are shown in Additional file 5: Figure S4 and Additional file 6: Movies S2-5. They illustrate the possible influence of orientation of the layer of fibers upon directionality of TEB elongation and growth since the TEB itself is angled away from the fiber layer with respect to its attached ductal structure. Interpretation of the type of collagen fibers based on SHG-B versus SHG-F detection, however, is limited in these images due to the “shadowing” artifact.

#### Optimization of Carmine red imaging

**Figure 10:**
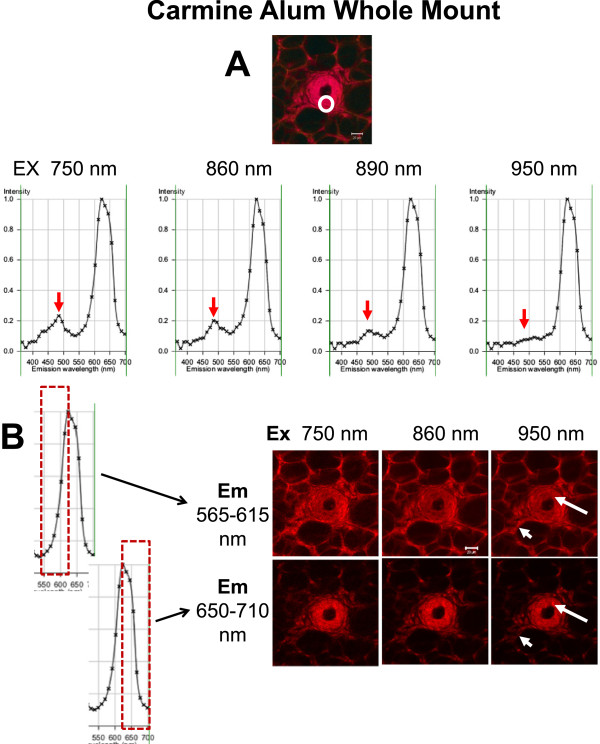
**Spectral emission scans at selected multiphoton excitation and effects of emission wavelengths: effects upon visualization of Carmine Alum-stained TEBs. A**. Whole mount mammary glands stained with Carmine Alum were imaged using 6 different multiphoton excitation wavelengths (Ex 750, 800, 830, 860, 890, and 950 nm) with laser power set at 0.1% in lambda mode to obtain 6 resulting spectral emission scans. The laser gain was altered to obtain good image intensity without saturation for each excitation wavelength. One major peak at Em 623 nm and a minor peak at Em 484 nm were observed. An image was generated from the Ex 860 nm lambda scan (Em 575–671 nm) including the peak emission at 623 nm. The white circle indicates the ROI used for the lambda scan at each excitation. **B**. Images were generated from Ex 750, 860, and 950 nm using two different emission wavelength ranges, Em 565–615 nm and Em 650–710 nm, representing the proximal and distal sides of the 623 nm peak (illustrated at left). At all excitation wavelengths, the images at the longer wavelength side of the peak have a better representation of epithelial tissue, at the expense of stroma. Arrows indicate differences in the images. Scale bars = 20 μm.

To improve upon the quality and to optimize and understand imaging of Carmine Alum fluorescence, we systematically explored excitation and emission conditions for detection of Carmine Alum. Lambda scans were obtained of a duct in cross section using excitation wavelengths 750, 800, 830, 860, 890, and 960 nm at 0.1% laser excitation power and adjusting laser gain to qualitatively compensate for the changes at different wavelengths (Figure 10A). Since the comparisons are not quantitative, the graphs of emission output are normalized in each case. These emission wavelength scans all produced a major peak at 623 nm emission (Figure 10A) with a minor peak at 484 nm and a very minor one at 431 nm, the latter obvious as a shoulder only at Ex 750 nm (Figure 10A, peak indicated by red arrows is 484 nm). An image was extracted from emissions 575–671 nm, representing a symmetrical sampling centered at the peak 623 nm using the “extract image” function in the Zeiss AIM software (not shown). The peak at 623 nm contains the majority of the Carmine Alum signal contained within the ductal epithelial cells compared with a similarly extracted image centered upon the minor peak at 484 nm (not shown). However, images extracted from bandwidths 565–615 nm favoured representation of the ECM and margins of the surrounding fat cells compared with images extracted from bandwidths of 650–710 nm which favoured nuclei of the ductal epithelium and fat tissue (Figure 10B, compare Em 565–615 nm and Em 650–710, long arrow indicates epithelium and short arrow matrix/stromal material).

**Figure 11:**
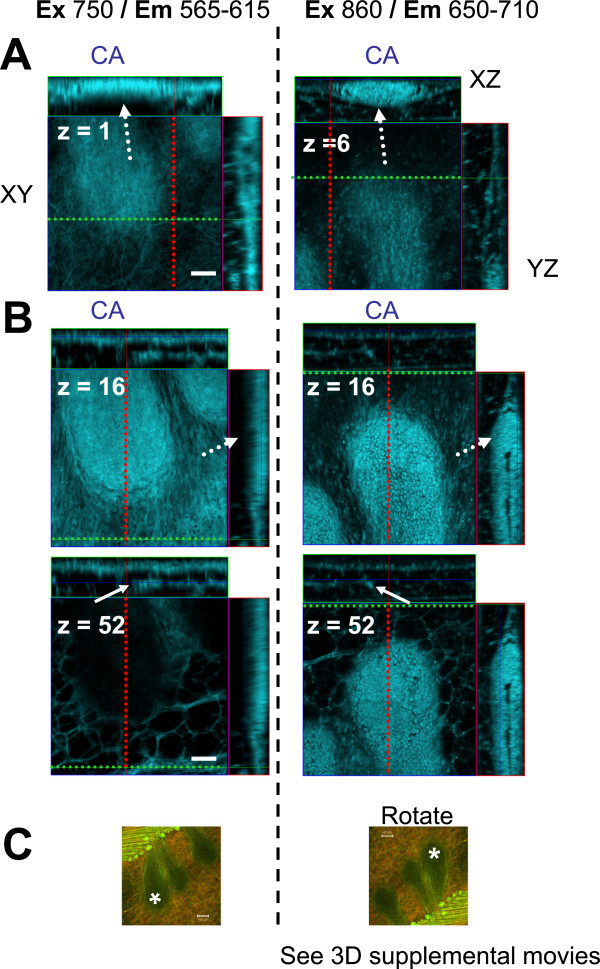
**Effect of emission wavelengths on the Carmine Alum image.** Orthogonal views of Carmine Alum images (from the experiment shown in Figures 4 whole mount and 8) are compared with the same TEB imaged using different settings. The left panels contain Carmine Alum images obtained using laser excitation set to 750 nm and emission wavelengths from 565–615 (BP 565–615 filter); the right panels contain Carmine Alum images obtained using laser excitation set to 860 nm and emission wavelengths from 650–710 (BP 650–710 filter). **A**. At left, Em 565–615 nm identifies primarily ECM/fibrillar layers and images the TEB epithelial cells poorly at increased depth (compare Z = 16 with Z = 52). At right, Em 650–710 nm identifies epithelial cells with improved imaging details at increasing z-depth. Dotted arrows identify the same position in the image. 3D movies are included in the Additional file (Additional file 6: Movies S4-5). **C**. Imaging orientation of the TEB is shown at low magnification (asterisk indicates the TEB imaged in **A**-**B**). **A**-**B**, Scale bars = 50 μm, **C**, Scale bars = 100 μm.

In all cases, at 750, 860 or 950 nm laser excitations, epithelial cell staining was isolated much more effectively and to greater Z-depths when emission bandwidths of 650–710 nm were used (Figure 11, Additional file 7: Figure S5, Additional file 8: Figure S6, Additional file 9: Figure S7). This phenomenon was confirmed by analysing a lambda scan of a duct at Ex 860 in which discrete bandwidths were used to extract images (Additional file 7: Figure S5A-B) which were then compared with images acquired at Ex 860 nm (in z-stacks) with fixed Em filters of Em 565–615 nm or Em 650–710 nm (Additional file 7: Figure S5C). Although the SHG-B and SHG-F signals did not change in either case, the appearance of matrix fibers and stroma was apparent in the former (Additional file 7: Figure S5B, Em 564–618 and Additional file 7: Figure S5C, Em 565–615) and not in the latter (Additional file 7: Figure S5B, Em 628–703 and Additional file 7: Figure S5C, Em 650–710).

A comparison of imaging depth was made using the two fixed bandwidth filters using Ex 860 and then the thresholded levels of Carmine Alum and SHG-B and SHG-F signals were quantified (Additional file 8: Figure S6). Whereas SHG-B and SHG-F signals were similar, the depth of penetration of the Carmine Alum signal was more robustly imaged when the Em 650–710 filter was used (Additional file 8: Figure S6B, dashed boxes). Despite using Em 650–710 at Ex 860 nm, Carmine Alum stained TEBs are indeed still “shadowed” at greater z-depths, whereas Carmine Alum stain present in fat cells was not affected to nearly the same degree at increasing Z-depths (Additional file 8: Figure S6B, compare CA 650–710 (FAT) trace in blue to CA 650–710 (TEB) trace in green). We conclude that the epithelial cell structure in particular could be highlighted at the expense of the stromal fibrillar material by selecting the emission wavelengths greater than Em 623 with the added advantage of optimizing the signal recovery of Carmine Alum at greater imaging depths. Another example of the degree of signal recovery obtained using Em 650–710 is shown in Additional file 9: Figure S7. The degree of heterogeneity in TEBs, their orientation, and their depth within the mammary gland makes aggregation of measurements for Em filter comparison very difficult. It is similarly difficult to assign an average Z-depth for successful signal recovery that would be typical for TEBs.

### Unstained whole mounts: characterization of autofluorescent and SHG signals

Whole mounts of fixed, unstained GFP and non-GFP mice were compared in order to assess whether the minor emission peaks observed in Carmine Alum lambda emission scans were due to the presence of GFP or autofluorescence (Additional file 10: Figure S8A). ROI’s selected in the ductal epithelium (Additional file 10: Figure S8A, green curve) were compared with ROI’s selected in the ductal space which would be predominantly background (Additional file 10: Figure S8A, red curve). Both GFP and non-GFP glands revealed a major peak at 495 nm in the normalized plots with little difference between the normalized emission curves and no peak coinciding with GFP. The background peak at 495 nm coincides with the background peak previously observed in Carmine Alum lambda scans (Figure 10A, arrows). Background intensity (red curve) was substantially less than the signal from the ductal epithelium when plotted as absolute value rather than normalized intensity (not shown). Background peaks were observed at 735, 860 and 960 nm with 860 nm producing the best signal-to-noise ratio for tissue contrast (Additional file 10: Figure S8B). Since the results were essentially the same for both GFP and non-GFP mice, we conclude that the GFP signal at 506 nm is lost after fixation and processing for unstained whole mounts, and the peak at 495 nm represents background autofluorescence derived from the unstained, but otherwise identically processed gland.

Images suitable for morphological studies were obtained at Ex 860 and 890 nm and included SHG-B and SHG-F signals (Additional file 11: Figure S9A). SHG-B was detected at Ex 800 and 890 nm (Additional file 11: Figure S9B), but not at Ex 735 nm (not shown), and could be separated from the background autofluorescence by using the lambda scan to generate narrow bandwidth images (Additional file 11: Figure S9B, green arrows and SHG-B images). However, SHG-F was observed only at Ex 860 and 890 nm and not at 800 nm (Additional file 11: Figure S9C). The ChD at Ex 800 nm and below captured only the DIC image (Additional file 11: Figure S9C, Ex 800, unfiltered, ChD). Increased signal-to-noise detection of fibers using SHG-B signals could be optimized in unstained whole mounts by narrowing the emission bandwidth to Em 393–425 as illustrated in a lambda scan, extracted emission images, and linescan quantification of pixel intensities (Additional file 12: Figure S10).

**Figure 12:**
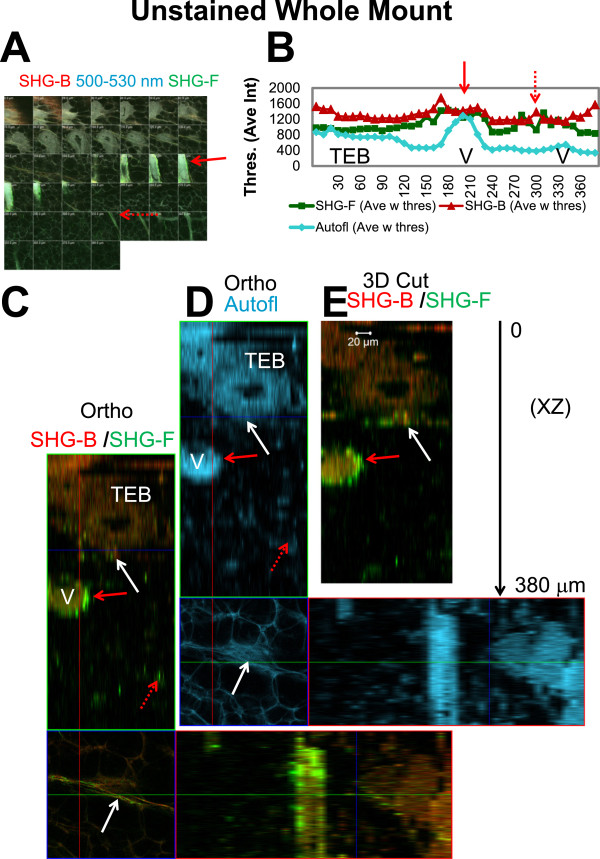
**Imaging unstained whole mount tissue. A**. An unstained mammary gland whole mount was imaged to a depth of 380 μm using SHG-B (Em 390-465 nm, red), autofluorescence (Em 500–530 nm, blue), and SHG-F (unfiltered ChD, green). **B**. Measurement of intensities from background subtracted and thresholded images demonstrate that SHG and autofluorescence were detected throughout the Z-stack and are an example of the imaging depth details obtained for tissue archived as unstained whole mounts. Thresholded average intensities of individual frames plotted against Z-depth are shown for the SHG-B (red), SHG-F (green) and autofluorescence (blue) channels. In **C** and **D**, orthogonal views, and in E, a vertical 3D “cut” view of the same sample, features are revealed at Z-depths directly below a TEB which is positioned near the gland surface. In **A**-**E**, red solid arrows indicate the vessel at medium Z-depth and dotted red arrows indicate SHG-B and SHG-F signals at a deeper Z-depth. The white arrows point out SHG-F and SHG-B signals directly below the unstained TEB. The SHG-F signal is brighter in association with vessels compared with TEBs. C-E. Scale bar = 20 μm.

Apart from retrospective studies of existing Carmine Alum prepared whole mounts, it is apparent from Z-imaging experiments that preparation of whole mounts, minus Carmine Alum staining, increases the depth of imaging by using autofluorescence of the gland avoiding Carmine Alum “shadowing” by TEBs. In unstained whole mounts, the mammary gland stromal details could be imaged to depths of ~340 μm beneath the unstained TEB, in this case without great loss of signal (Figure 12A-D). Significantly, SHG-B and SHG-F signals appear not to be affected to the same extent by the presence of a Carmine Alum stained TEB; that is, the unstained TEB appears not to shadow the SHG signal in the same way as observed for the Carmine Alum stained TEBs (Figure 12C-E). Figure 12E is a 3D cut showing SHG-B and SHG-F signal. The white arrows in Figure 12C and E illustrate the SHG-B and SHG-F signals below the TEB, deeper in the tissue. In areas lacking TEBs, muscle tissue could be imaged at z depths of up to ~ 200 μm and possibly deeper, although this was not investigated in detail (Additional file 13: Figure S11A-E). We conclude that dense Carmine Alum staining in TEBs itself is largely responsible for the “shadowing” effect below TEBs. The penetration depended in large part on the presence and specific relative location of Carmine Alum-dense ducts and TEBs interposed between light source and detector (Additional file 4: Figure S3).

## Discussion

Using a multiphoton microscope modified by the replacement of the transmitted detector with a non-descanned detector, we have demonstrated a convenient method to image Carmine Alum staining simultaneously with reflected and transmitted SHG signals. Using a single laser excitation set to 860 nm, this method allows imaging of ductal and terminal end bud structures in whole mount preparations without significant tissue rearrangement following preparation and using common bandpass filters. This method enhances the imaging of epithelial cells at the expense of stromal tissues when an emission bandpass filter BP 650–710 is used. Carmine Alum has been reported to bind most intensely to nuclei [28,29] and it may be that this can best be appreciated using the longer emission bandwidths and is the explanation of why epithelial ductal tissue is visually enhanced since nuclear density is higher in that tissue compared with the stroma. Emissions collected at 565–615 include background autofluorescence from all of the fixed tissue including extracellular matrix and stroma. Thus, an alternate goal might be to use emission BP 565–615 to simultaneously image stromal and epithelial morphology by combining the Carmine Alum signal with the autofluorescent signal. This appears to be the probable mechanism of the emission wavelength dependence of Carmine Alum staining. In summary, the Carmine Alum fluorescence imaging procedure can be flexible, since it could be accomplished at many different laser excitations and filter combinations; it can be accomplished using a conventional confocal detector if a non-descanned detector is unavailable, and the emission peak is strong and relatively broad. In addition to selectively imaging Carmine Alum emission signals, simultaneously imaging SGH-B and SHG-F signals reveals new information on ECM fiber structure and orientation relating to TEB and ductal structure.

### Mammary gland architecture

Live imaging of the GFP mammary gland demonstrated an outer mammary gland layer of strong SHG-B signal characterized by increasingly parallel fiber orientation with Z-depth, and high resolution profiles of GFP-tagged epithelial cells. However, transmitted SHG-F signals were highly scattered resulting in an apparently “shadowed” pattern matching the outline of fat cells within the stroma. At face value, the data suggest a layer of fibers that is approximately 6 μm thick on the surface of the mammary gland, but nothing deeper within the tissue. However, unstained whole mount preparations or those stained with Carmine Alum afforded much more detailed structural information from deeper within the mammary gland. Whereas separate thin layers of oriented fibers could be detected in areas immediately adjacent to TEBs in living tissue near the surface, extensive layers of fibers were detected in whole mount mammary glands. Furthermore, SHG-F signals deeper within the tissue became apparent after whole mount tissue preparation with or without Carmine Alum staining. We conclude that layers of fibers delineate zones that isolate TEBs and ductal growth from surrounding stromal layers.

The fibrillar layers contain both reflected SHG-B and transmitted SHG-F signals. A similar mixture of these signals is found where the fibers run in parallel orientations to the vessels. The SHG signals associated with blood vessels are significantly stronger. Subdivisions of collagen layers within the stroma might divert the directionality of ductal extension by interacting with TEBs, as suggested by images such as Additional file 5: Figure S4, and Additional file 6: Movies S2-3. Dense fibrillar structure is associated with intense ductal bud formation in abnormal glands illustrated in Figure 7. These details were only obtained using a combination of SHG-B and SHG-F. Thus, a combination of reflected and transmitted SHG images together with visualization of ductal structures and TEBs stained with Carmine Alum reveal additional architecture in 3D within mammary glands not previously appreciated.

### Extracellular matrix

Previously, SHG signals visualizing fibrillar structures have been identified as primarily due to fibrillar collagen I by means of antibody, collagenase, and *in vitro* experiments (for example, [22,30]). Thus, we conclude that the fibrillar structure identified using SHG in these studies are likely collagen I fibrils. Furthermore, using SHG-B, collagen I fibers have been shown associated with TEBs, and their abundance and rigidity demonstrated to be controlled by macrophage activity [6,22,31]. Using an imaging platform with both coherent anti-Stokes Raman scattering and second harmonic generation, Le et al. imaged mammary adipocytes, blood capillaries, collagen fibrils, and tumor cells simultaneously and without any labelling [32]. They observed that lipid droplets of adipocytes and collagen content in mammary tumor stroma were both increased in obese animals, measurements that could only have been made by imaging tissue in 3D, and not possible with 2D histology. Although autofluorescence of collagen has been used to measure collagen density in multiphoton experiments [33], the ability to image and quantify both the ductal architecture and density of collagen fibers in archived mammary gland whole mounts provides a simple but powerful tool for mammary gland biology. The evaluation of collagen fibril arrangement and density for breast cancer prognosis was recently reported [8]. The imaging was carried out on archived pathology tissue sections. To understand the basis for the association of collagen structure to prognosis observed in human tissues, studies of animal models of breast cancer are critical. In the present study, we have determined that archived Carmine Alum-stained whole mount tissues are candidates for multiphoton imaging to explore collagen fiber deposition in transgenic animal models of cancer. Molecular, cellular, and tissue imaging has and will continue to provide valuable information on the role of collagen deposition in normal and tumor development.

### SHG

Zoumi et al. optimized SHG of 3D collagen raft cultures and found peak SHG-B at approximately 800 nm [21]. We found that although 860 nm may not be optimal for SHG, the signal was adequate to provide good images of collagen fibers, and at the same time useful for either GFP imaging (in live tissue) or Carmine Alum (in whole mounts). Imaging depth would likely be improved by imaging at 950 nm excitation with a SHG signal appearing at 475 nm. However, Zoumi et al. observed that the reflected SHG signal declines dramatically and is quite low even at 880 nm [21]. Therefore, use of the SHG-F signal might be preferable when imaging at longer wavelengths such as 960 nm is required. On the other hand, Theodossiou et al. (2006) reported for rat-tendon cryosections that SHG-F signal peaked at 880 nm with secondary maxima about 845, 895, and 915 nm, whereas SHG-B signal had four maxima at 845, 880, 895 and 915 nm [34]. Thus, it is possible that MP excitation at 880 nm would provide sensitivity for both SHG and EGFP [34,35]. These authors also reported that as the fiber orientation angle with respect to the laser directionality increased from 0 to 90 degrees, SHG intensity also increased. The orientation of the mammary gland on the stage ensures that many of the fibers would be mostly perpendicular to the laser source as the terminal end buds and their associated parallel fibers are mostly perpendicular as well, and thus their intensity would be maximized. However, fibers that are oriented parallel to the laser source would be masked. Indeed, in orthogonal views, fibers are never seen in vertical arrays, but rather as punctate cross sections. To answer this question completely, the tissue must be rotated 90^o^ so that the fibers are reoriented parallel to the laser beam.

As previously described [23], ECM fibers in the mammary gland visualized using SHG-B appeared to be somewhat more diffuse and irregular in profile compared with the fibers seen using SHG-F (see Figure 9). Previously, the ratio of SHG-F/SHG-B signal was found to change when comparing immature to mature collagen fibers [23]. Examination of the punctate or irregular aspect of reflected SHG signal was therefore proposed as an indicator of the presence of immature fibers. According to this interpretation, in our study, the SHG-B signals identify collagen fibers of more variable fiber maturity compared with the SHG-F signals which would suggest that SHG-F-detected fibers within the gland might have a more crystalline or mature structure. The verity of this is hard to assess. Carmine Alum staining might interfere in some way with SHG detection in addition to reducing signal intensity via a “shadowing” effect. However, this is unlikely since fluorescence and SHG detection occurs by two different mechanisms, and Carmine Alum poorly stained the fibers. Furthermore, SHG-B and SHG-F signals were detected in similar arrangements in unstained, whole mount mammary glands. Another interesting possibility is that whole mount preparation involving fixation and dehydration might actually improve SHG intensity not only by defatting the gland, but also by stabilizing or unmasking the repeating structure of collagen fibrils, especially in the case of SHG-F that was poorly detected *in vivo*. Thus, further studies are required to examine the utility of the SHG-F compared with SHG-B signals to reveal differences in collagen fiber properties and for quantitative comparison of SHG-detected fibers.

### Limitations and considerations for imaging

Imaging morphology in Carmine Alum-stained mammary gland whole mounts is limited in that the dense array of Carmine Alum-stained epithelial cells in terminal end buds scatters both the fluorescence and SHG signals depending upon imaging depth and the relative positions of the epithelial or collagen fiber structures with respect to the detectors. Study of unstained whole mounts suggests that the “shadowing” effect of TEBs upon SHG and Carmine Alum signals deeper within the tissue likely arises from the density of Carmine Alum staining itself. In any case, the investigator must pay careful attention to the surrounding structures before interpreting the structural data. Another potential limiting factor to acquiring deep tissue images in whole mounts is the working distance of the imaging lens. TEBs deeper into the whole mounts are accessible with long working distance lenses in the case that there are no intervening epithelial structures between the TEB chosen for imaging and the coverslip surface.

For deepest imaging into the whole mount tissue, it is probably advisable to collect the Carmine Alum signal using the higher emission wavelengths above 623 nm to detect the epithelial cells and eliminate ECM/ stromal contribution from autofluorescence (see Figure 11). At higher excitation and emission wavelengths, tissue penetration would be enhanced in addition. Increasing laser intensity might be important to gain depth of SHG signal and certainly using a non-descanned detector for SHG-B would be optimal. However, if higher intensity laser illumination is used, Carmine Alum should be imaged first at low laser intensity to minimize photobleaching and photodamage. In cases where the researcher is interested to compare SHG-B and SHG-F, appropriately narrow bandpass filters (or ranges) for both reflected and transmitted signals should be employed to block out autofluorescent signal. In the system used for this study, the META detector and collection into ChS can be used to limit the SHG-B to a narrow range around the peak emission to improve the signal to noise of the SHG-B detected collagen fibers and the emission range for Carmine Alum selected to exclude background signal that occurs overlapping with wavelengths at least up to 623 nm (see Additional file 11: Figures S9B and Additional file 12: S10A-C).

Maximal collection of photons is critical to maximize the depth of imaging that is possible, and to do this a non-descanned detector should be used rather than the descanned, confocal detector. In this study, use of a non-descanned detector to collect the SHG-B and Carmine Alum signals was not possible. However, a non-descanned detector with greater sensitivity than the transmitted detector did allow imaging of SHG-F. The presence of high quality non-descanned detectors to maximize photon collection for both transmitted and reflected fluorescence and SHG emissions, would likely increase z-depth detail, even though the operator would also requires a more elaborate set up to minimize stray light that is present even in a darkened room.

Finally, it must be kept in mind that artifacts arising from whole mount preparation could have negative effects on relative positions of fibers, TEBs and vessels, particularly if the investigator is not careful to minimize or at least monitor size and shape changes in the gland prior to imaging. In particular, the tissue should be stretched to approximate the *in situ* shape and size and then mounted between two large, 0.17 mm thick coverslips for imaging. This allows the entire whole mount to be flipped over and the same TEBs imaged again, but in the reverse orientation with respect to reflected and transmitted light. A fiducial mark could be introduced using MP laser photodamage at a particular XYZ site to facilitate re-orientation.

## Conclusions

In conclusion, multiphoton excitation microscopy coupled with second harmonic generation imaging can be successfully used to determine mammary gland architecture in unstained or Carmine Alum stained whole mounts. Layers of collagen fibers detected using a combination of SHG-B and SHG-F define zones within the mammary gland in which elongating TEBs reside during gland maturation. Abnormalities in TEB development undetected by bright field viewing of Carmine Alum whole mounts can be detected using multiphoton imaging as a precursor or replacement for paraffin embedding and sectioning. Careful mammary gland whole mount preparation including stretching the gland to an extent comparable to what was experienced *in situ* allows deeper imaging into the tissue than is possible with live material and detection of SHG and fluorescent signals several hundred micrometers deep.

## Competing interests

Both authors declare that they have no competing interests.

## Authors’ contributions

SCM performed the majority of the imaging experiments and wrote the manuscript. MJ participated in manuscript preparation, and in performing or supervising the animal experiments and preparations. Both authors read and approved the final manuscript.

## Pre-publication history

The pre-publication history for this paper can be accessed here:

http://www.biomedcentral.com/1471-2407/13/373/prepub

## Supplementary Material

Additional file 1: Figure S1 Simplified method to image mammary gland whole mounts using reflected and transmitted SHG together with Carmine Alum fluorescence at a single excitation wavelength, 860 nm. A. Zeiss AIM software images are presented in lieu of a diagram to illustrate the light path and filter combinations. At left, the configuration control contains red lettering to label the filter icons. In the image view containing details of the filters at right, the red letters are associated with filter details. B. A single image plane is shown with its associated orthogonal slices above (XZ) and to the right (YZ). Dashed lines indicate planes of associated images; red for YZ, green for XZ, and blue for XY. Multiple SHG positive fibrillar layers are present (arrows). Scale bars = 50 μm. Click here for file

Additional file 2: Figure S2 Multiphoton and SHG spectral emission scans using live GFP-mouse tissue. An isolated GFP mouse mammary gland was imaged in the lambda mode (spectral emission) using a Zeiss LSM510/META/NLO with MP excitation of 860 nm and a 25X/0.8 N.A. Zeiss lens. A. At left, the graph represents emission wavelength (X axis) plotted against average intensity (Y axis). At right, the profile of the mammary gland TEB is shown using the “lambda coded” setting. B. The graph at left indicates the emission wavelength profile for three ROIs shown in the lambda coded image at right. Higher magnification views of the ROI area are indicated by arrows and shown as insets. C. Extracted images with emission bandwidths of Em 404–446 nm, 446–478 nm, 500–532 nm, and 596–703 nm. Images of the SHG-B and GFP peaks at 431 nm and 506 nm, respectively, are in the first and third images. GFP positive cells are indicated by the arrow. Scale bars = 50 μm. Click here for file

Additional file 3: Movie S1 Z-stack movie of abnormal TEB from HAI-1 gland. The image stack of 1 μm sections totaling 74 μm was used to generate the 3D and orthogonal views shown in Figure 6, is presented as a QuickTime movie. The abnormal lumen near the tip of the TEB is not continuous with the main ductal lumen. Click here for file

Additional file 4: Figure S3 Scattering artifacts on SHG signals generated in thick specimens. Single XY and orthogonal images illustrate the variable ability of SHG signals to reach the detector. A. The Carmine Alum stained TEB overlying the fibers associated with several blood vessels prevent the transmitted SHG signal (green) reaching the ChD, NDD detector and illustrated in the diagram (compare SHG-B/SHG-F images with SHG-B/CA/SHG-F images). B. A single vessel detected by SHG-B (red) is closest to the coverslip. A TEB deeper into the tissue contains a gradient of blue Carmine Alum fluorescence to green (the latter color due to the background Carmine Alum staining included in the SHG-F detector). C. Loss of reflected signals occurs when the SHG-B source is deeper within the tissue relative to the TEB, and the TEB blocks the return of the reflected signal to the internal detector. Scale bars = 50 μm. Click here for file

Additional file 5: Figure S4 Three dimensional (3D) representations of TEBs. Z-stack of images 178 μm deep was collected to include a TEB from a Carmine Alum whole mount. A. A 3D reconstruction was made using the Zeiss AIM software which illustrates a sheet of mostly parallel collagen fibrils visualized exclusively by SHG-F (green, dashed boxed area). The TEB is oriented so that the vector followed by the TEB is at a different angle than the vector along which the attached duct lies (arrows). The QuickTime movie of the Z-stack is shown in Additional file 6: Movie S2, and the subregion indicated by the dashed box area is shown in Additional file 6: Movie S3. B. A single Z-slice at a depth of 32 μm reveals the close apposition of SHG-F positive fibers to the TEB caught in an oblique section. C. A 3D reconstruction of the same Z-stack shown in A was made using Volocity software and the transparency chosen to best illustrate the layer of fibers detected by SHG-F (green) in contact with the tip of the TEB. The view looking towards the TEB from the perspective of the coverslip (0^o^) compared with the view from the opposite direction (180^o^) demonstrates that the layer of SHG-F-detected fibers (green) lies deeper within the tissue and is most easily visualized from the 180^o^ perspective. D. The image stack used to illustrate Figures 4, 8, and 11 was subjected to 3D reconstruction using Volocity software. A QuickTime animation of the Z-stack is found in Additional file 6: Movie S4. Additional file 6: Movie S5 is a QuickTime animation of the 3D views. In various rotations, the association of layers of fibers with the TEBs is shown, with SHG-B signal predominating at the near surface of the imaged TEB (red) and SHG-F signal predominating at the far surface (green). A-C, Scale bars = 50 μm. Click here for file

Additional file 6: Movies S2-S5 Association of SHG signals from fibers associated with TEBs. Movies 2–5 are QuickTime animations from Additional file 5: Figure S2 A (Movies 2–3) and C (Movies 4–5) and include Z-stack animations (Movies 2 and 4) and 3D animations (Movies 3 and 5). Click here for file

Additional file 7: Figure S5. Effects of emission bandwidth collection on Carmine Alum/ SHG-B/ SHG-F images. A. A lambda scan of a Carmine Alum stained TEB is illustrated together with a graph containing data from three ROIs (blue-background, red-ductal cells near tip of TEB and green-material associated with the TEB margin). B. Images were extracted with defined wavelengths using the Zeiss Meta software. EM 628–703 nm identifies primarily the epithelial tissue whereas the Em 564–618 nm extracted image is striking in the appearance of the TEB-associated material, fat cell outlines, and the shadowing artifact on the TEB (arrow), C. 3D Carmine Alum images (blue) of the same TEB were obtained using Ch3 and fixed emission filters of Em 565–615 (at left) and Em 650–710 (at right) together with SHG-B (red) and SHG-F (green) 3D images. Scale bars = 100 μm. Click here for file

Additional file 8: Figure S6 Quantification of data from Additional file 7: Figure S5C. A-B. To determine the changes in intensity for SHG-B, Carmine Alum, and SHG-F, image stacks were background subtracted and thresholded for Carmine Alum-stained structures, and fibers within the SHG-B and SHG-F images and the average intensity per ROI plotted. ROIs included the entire frame, and the ROIs indicated by the ROI boxes in A over a TEB (TEB) and an area of the stroma containing fat cells (Fat). B. Graphs for each color plane as indicated and Em 565–615 nm (at left) and Em 650–710 nm (at right) are presented. Dashed boxes in the Carmine Alum graphs invite comparison of the loss of stain intensity in the TEB with increasing Z-depth (green trace) in the Em 565–615 nm trace at left relative to the Em 650–710 nm trace at right: improved intensity with increasing Z-depth was observed for Em 650–710 nm. Imaging was identical with the exception of bandpass filter selection. Click here for file

Additional file 9: Figure S7 Quantification of “Shadowing” in Carmine Alum Wholemounts. The TEB presented in Additional file 6: Movie S3 and Additional file 5: Figure S4 A-C was used to quantify the loss of Carmine Alum signal with imaging depth within a TEB. A. Orthogonal views illustrating this Carmine Alum-stained TEB. B. Single plane image illustrating the sites of ROIs for analysis (yellow boxes over TEB and Fat within the stroma). C. Graphs of the data by thresholded area and average intensity. The dotted arrow indicates the midpoint of the TEB where the lumen space contributes to the dip in thresholded intensity. Average intensity drops off beyond the midpoint of the TEB at aprroximtely Z = 90 μm. A, Scale bars = 50 μm. Click here for file

Additional file 10: Figure S8 Comparison of Emission Spectra of GFP and non-GFP Unstained Whole Mount Tissue. A. GFP mice and non-GFP mice were prepared for whole mount without Carmine Alum staining and a lambda scan obtained from Em 361–704 nm using Ex 860 nm. ROI’s in a background area and one within the ductal epithelium were graphed as a function of normalized intensity (arrows indicate insets of ROIs at higher magnification). The intensity scale is normalized for comparison of ROIs. B. GFP mice were prepared for whole mount without Carmine Alum staining and lambda scans were obtained from Em 361–704 nm at Ex 890, 860, and 735 nm. ROI’s in a background area and one within the ductal epithelium were graphed as a function of relative intensity (arrows indicate insets of higher magnification). Average intensity of each ROI is plotted. Scale bars = 100 μm. Click here for file

Additional file 11: Figure S9 Comparison of excitation wavelengths for imaging unstained whole mount tissue. A. SHG-B and SHG-F (unfiltered) images were obtained of a single XY plane at a region rich in fibers (upper panels) from a GFP-mouse. At a deeper XY plane that included a median section through a TEB, the autofluorescent background signal was included in a three-color image (lower panels). Comparison of the images at Em 860 and 890 nm reveal little difference in the qualitative information present. B. SHG-B was detected within a focal plane including fibrillar structures and was compared for Ex 735, 800, and 890 nm. None was detected for Ex 735 nm (not shown). An ROI was selected for a region containing fibrils (red circle, red arrow on the lambda coded image) and the average emission intensity plotted for each excitation wavelength. An extracted image was obtained for emission wavelengths containing the SHG maximum intensity: for Ex 800 nm the peak was 400 nm and for Ex 890 nm, the peak was 445 nm (SHG-B, green arrows). C. The transmitted signal was detected for Ex 890 (Ex 890, ChD), but was not detected for Ex 800 or Ex 735 (not shown). A DIC image appears using Ex 800 in ChD, whereas SHG-F appears at Ex 890 (yellow asterisk). Scale bars = 50 μm. Click here for file

Additional file 12: Figure S10 Reduction in background fluorescence with narrowed SHG-B emission bandwidth. A. A lambda scan was obtained from Em 361–704 nm using Ex 860 nm. A subset of wavelengths is illustrated where the blue arrows indicate the SHG-B signal (420 and 431 nm). B. Bandwidth images were extracted using Zeiss Meta software; blue arrows indicate the SHG-B fibers seen in A. C. Linescans of pixel intensity performed indicated by dashed lines on the two images in B demonstrate the increased signal to noise detection of the fibers (blue arrows). The same TEB was imaged also in Additional file 11: Figure S9A. Click here for file

Additional file 13: Figure S11 Imaging unstained whole mounts containing muscle. A-D. A z-stack was obtained for unstained whole mount tissue at Ex 860 with Em 390–465 nm (A, red, SHG-B), Em 500–530 nm (B, blue, autofluorescence), ChD unfiltered (C, green, SHG-F), and brightfield (D). E. The average frame intensity of background subtracted z-stack images is graphed. The asterisk indicates the position of the SHG-B and SHG-F signal observed at 140 μm z-depth in A-C and E. Click here for file
