# Supplementary material for: Three dimensional multiphoton imaging of fresh and whole mount developing mouse mammary glands
Source: BMC Cancer. 2013 Aug 6;13:373. doi: 10.1186/1471-2407-13-373 (PMC3750743; doi:10.1186/1471-2407-13-373)
Supplement: Additional file 1: Figure S1 — Simplified method to image mammary gland whole mounts using reflected and transmitted SHG together with Carmine Alum fluorescence at a single excitation wavelength, 860 nm. A. Zeiss AIM software images are presented in lieu of a diagram to illustrate the light path and filter combinations. At left, the configuration control contains red lettering to label the filter icons. In the image view containing details of the filters at right, the red letters are associated with filter details. B. A single image plane is shown with its associated orthogonal slices above (XZ) and to the right (YZ). Dashed lines indicate planes of associated images; red for YZ, green for XZ, and blue for XY. Multiple SHG positive fibrillar layers are present (arrows). Scale bars = 50 μm. [file 1471-2407-13-373-S1.pdf]

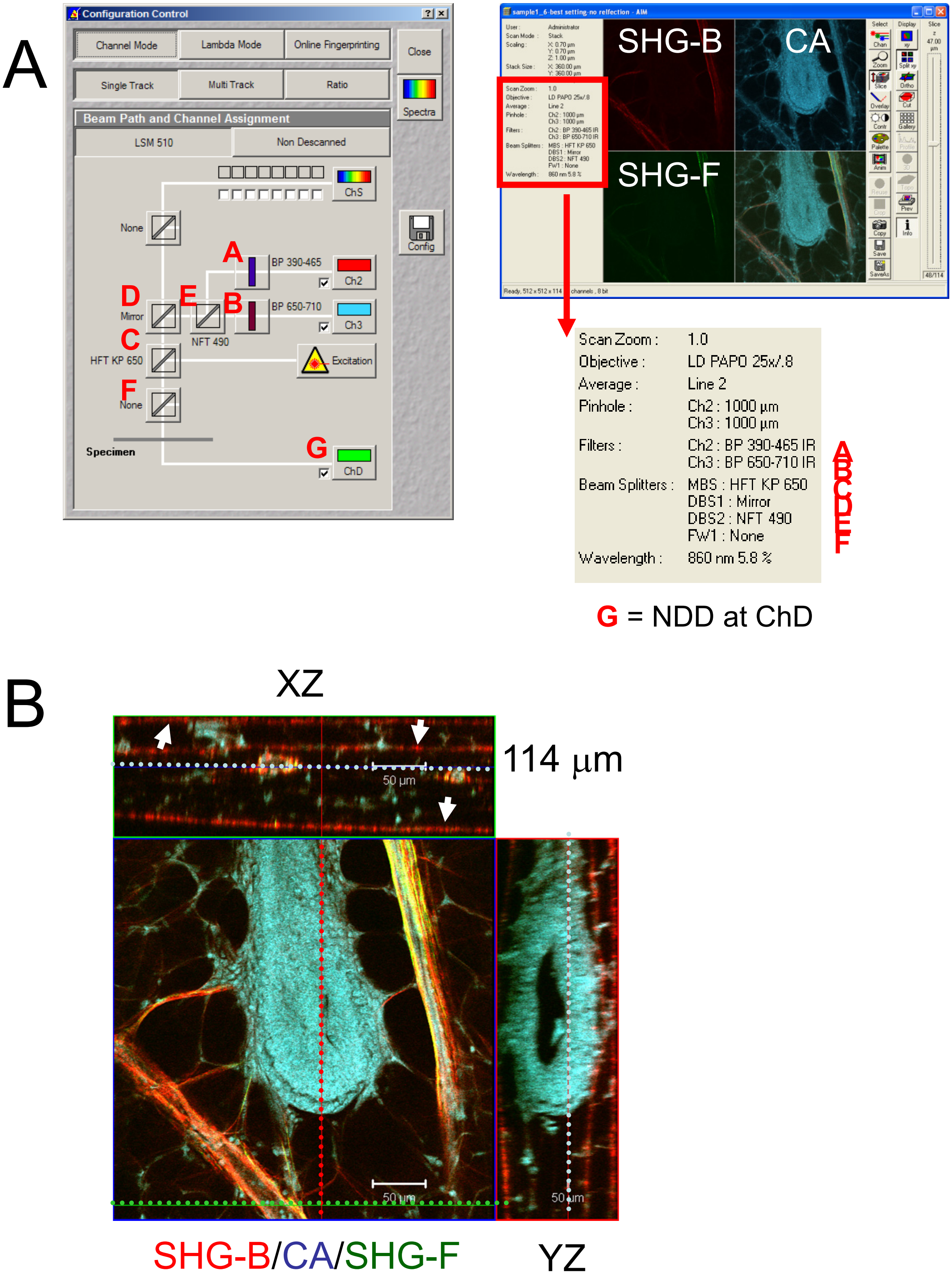
A
Configuration Control
Channel Mode
Lambda Mode
Online Fingerprinting
Close
Single Track
Multi Track
Ratio
Spectra
Beam Path and Channel Assignment
LSM 510
Non Descanned
ChS
None
Config
A
BP 390-465
Ch2
D
E
B
Mirror
BP 650-710
Ch3
C
NFT 490
HFT KP 650
Excitation
F
None
Specimen
G
ChD
SHG-B
CA
SHG-F
Scan Zoom : 1.0
Objective : LD PAPO 25x/.8
Average : Line 2
Pinhole : Ch2 : 1000 µm
Ch3 : 1000 µm
Filters : Ch2 : BP 390-465 IR
Ch3 : BP 650-710 IR
Beam Splitters : MBS : HFT KP 650
DBS1 : Mirror
DBS2 : NFT 490
FW1 : None
Wavelength : 860 nm 5.8 %
A
B
C
D
E
F
G = NDD at ChD
B
XZ
50 µm
114 µm
50 µm
50 µm
SHG-B/CA/SHG-F
YZ
