# Supplementary figures and images for: Three dimensional multiphoton imaging of fresh and whole mount developing mouse mammary glands
Source: BMC Cancer. 2013 Aug 6;13:373. doi: 10.1186/1471-2407-13-373 (PMC3750743; doi:10.1186/1471-2407-13-373)

# Live Tissue (Ex 860 nm)

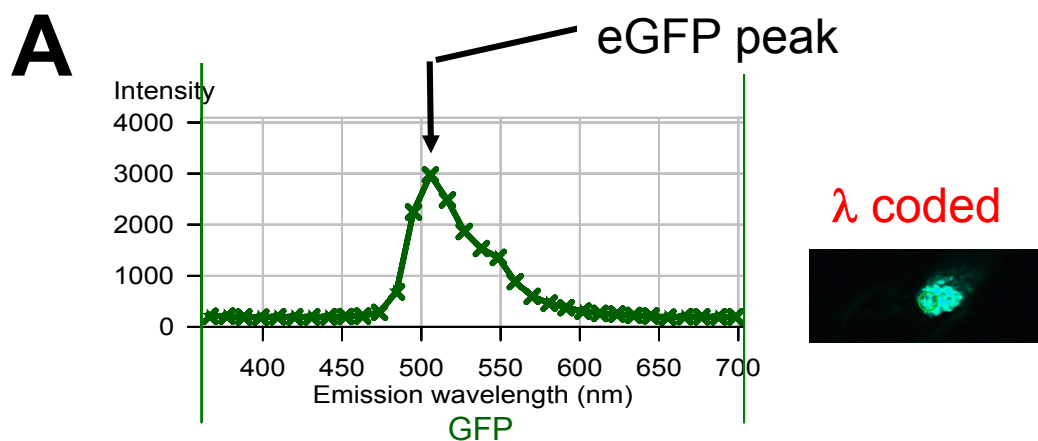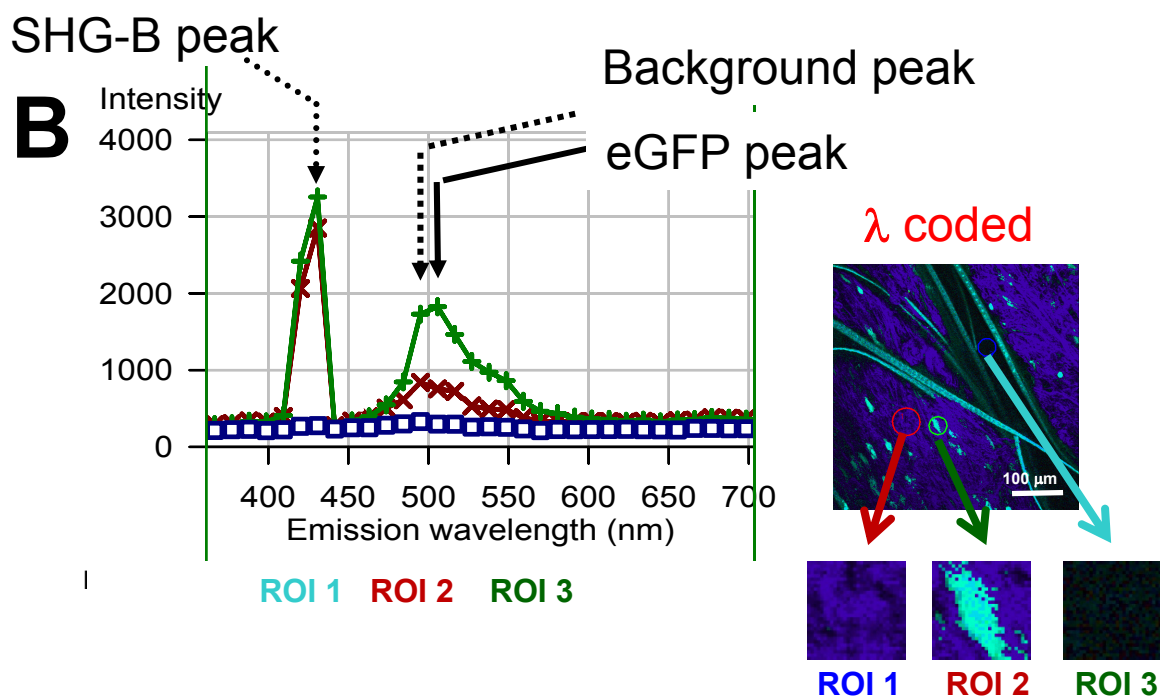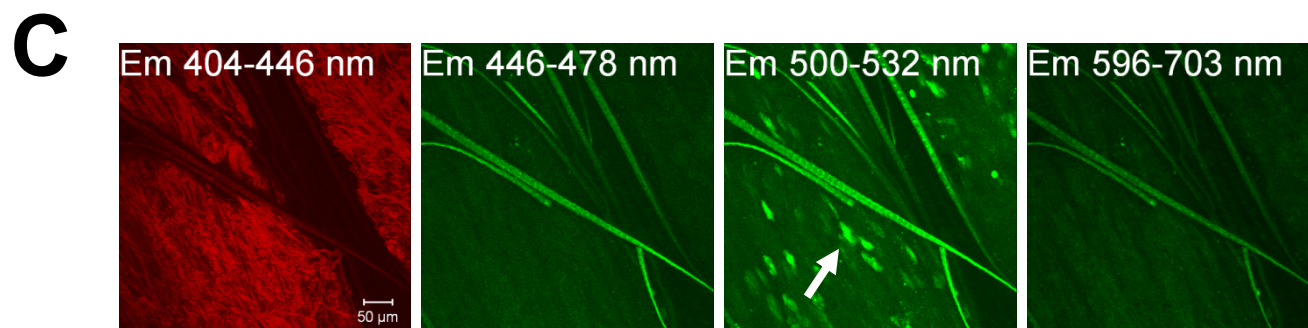

Supplement: Additional file 2: Figure S2 — Multiphoton and SHG spectral emission scans using live GFP-mouse tissue. An isolated GFP mouse mammary gland was imaged in the lambda mode (spectral emission) using a Zeiss LSM510/META/NLO with MP excitation of 860 nm and a 25X/0.8 N.A. Zeiss lens. A. At left, the graph represents emission wavelength (X axis) plotted against average intensity (Y axis). At right, the profile of the mammary gland TEB is shown using the “lambda coded” setting. B. The graph at left indicates the emission wavelength profile for three ROIs shown in the lambda coded image at right. Higher magnification views of the ROI area are indicated by arrows and shown as insets. C. Extracted images with emission bandwidths of Em 404–446 nm, 446–478 nm, 500–532 nm, and 596–703 nm. Images of the SHG-B and GFP peaks at 431 nm and 506 nm, respectively, are in the first and third images. GFP positive cells are indicated by the arrow. Scale bars = 50 μm. [file 1471-2407-13-373-S2.pdf]

**A**

XY

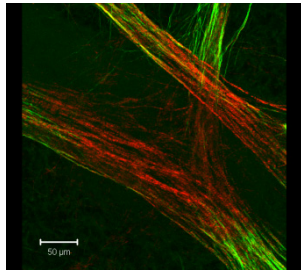

XZ

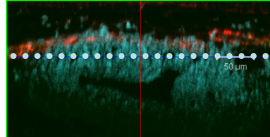

YZ

XY

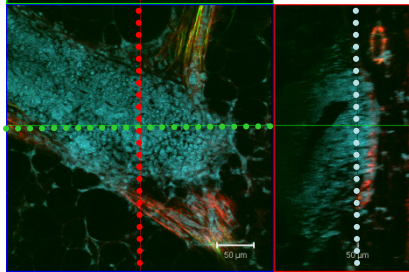**B**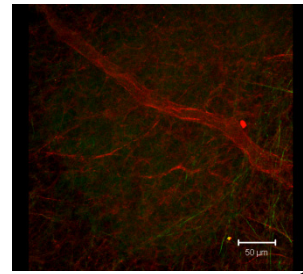SHG-B/  
SHG-F

Z = 0

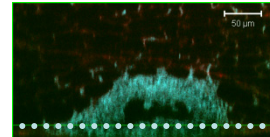SHG-B/  
CA/  
SHG-F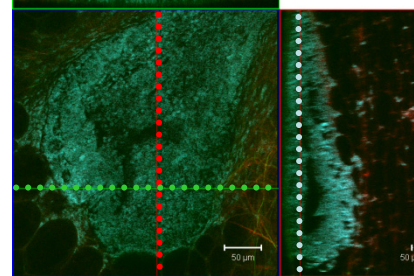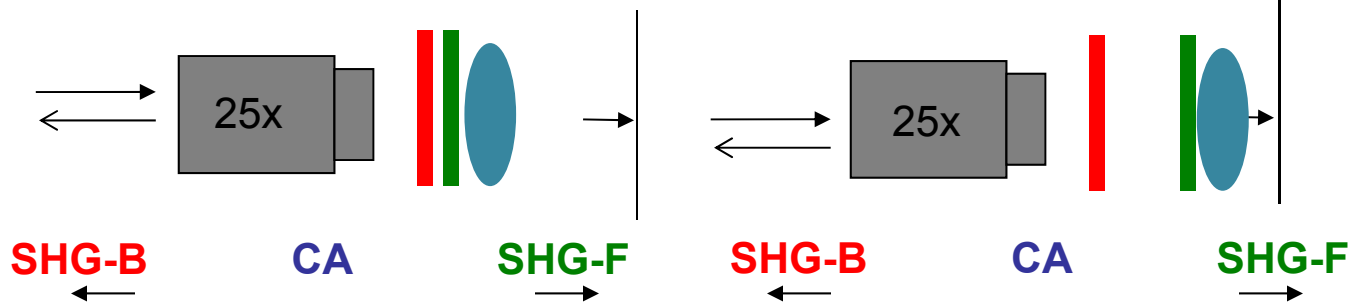**C**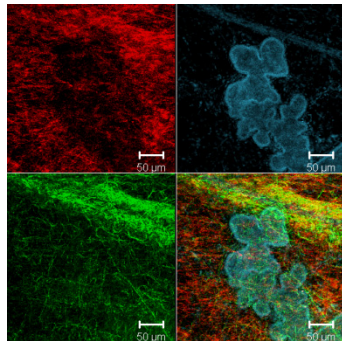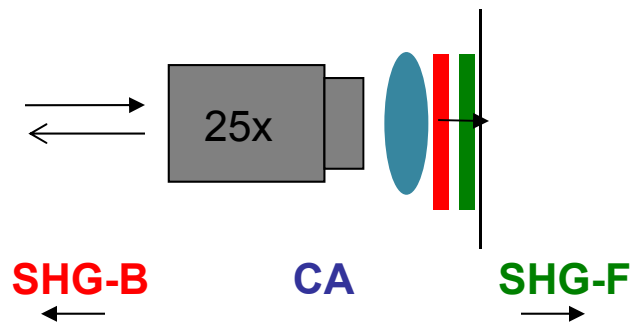

Supplement: Additional file 4: Figure S3 — Scattering artifacts on SHG signals generated in thick specimens. Single XY and orthogonal images illustrate the variable ability of SHG signals to reach the detector. A. The Carmine Alum stained TEB overlying the fibers associated with several blood vessels prevent the transmitted SHG signal (green) reaching the ChD, NDD detector and illustrated in the diagram (compare SHG-B/SHG-F images with SHG-B/CA/SHG-F images). B. A single vessel detected by SHG-B (red) is closest to the coverslip. A TEB deeper into the tissue contains a gradient of blue Carmine Alum fluorescence to green (the latter color due to the background Carmine Alum staining included in the SHG-F detector). C. Loss of reflected signals occurs when the SHG-B source is deeper within the tissue relative to the TEB, and the TEB blocks the return of the reflected signal to the internal detector. Scale bars = 50 μm. [file 1471-2407-13-373-S4.pdf]

**A**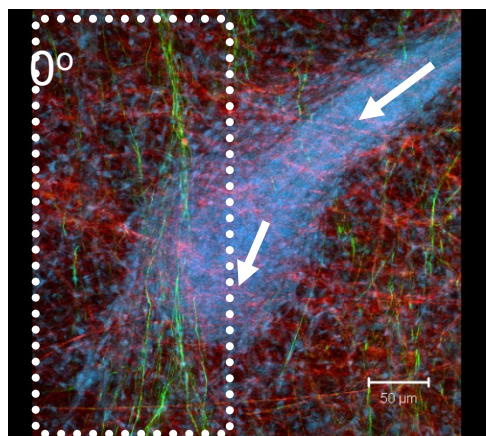**B**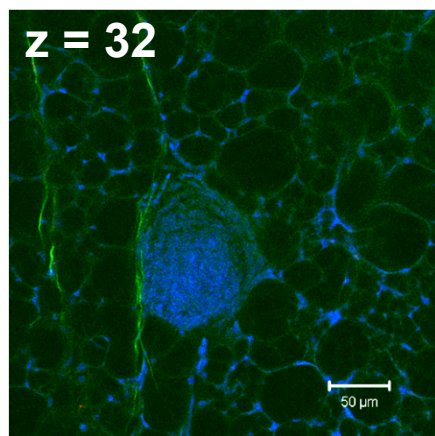

SHG-B/  
CA/  
SHG-F

**3D rotation movie****C**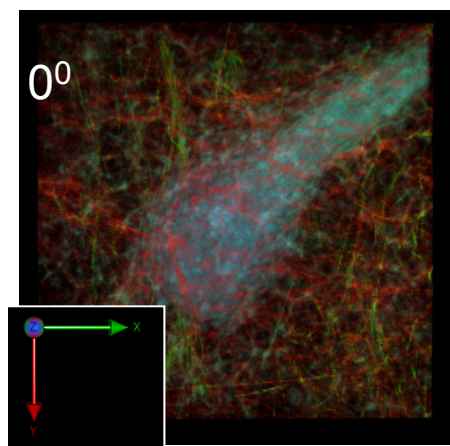**D**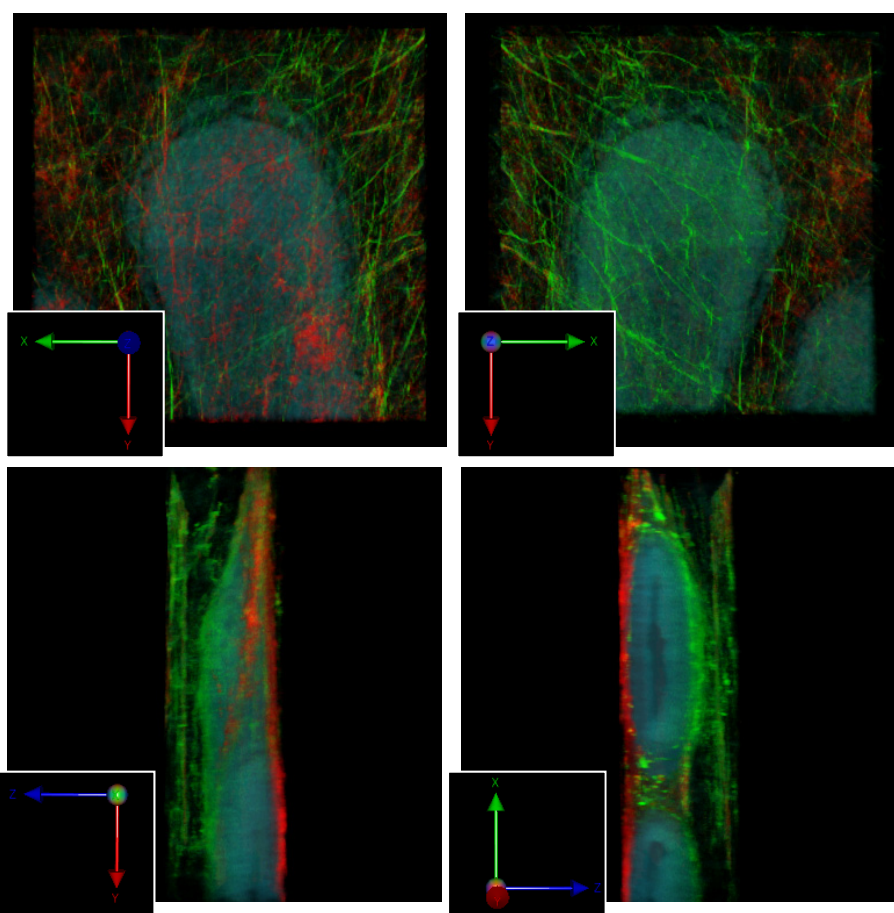**3D rotation and Z-stack movies**

Supplement: Additional file 5: Figure S4 — Three dimensional (3D) representations of TEBs. Z-stack of images 178 μm deep was collected to include a TEB from a Carmine Alum whole mount. A. A 3D reconstruction was made using the Zeiss AIM software which illustrates a sheet of mostly parallel collagen fibrils visualized exclusively by SHG-F (green, dashed boxed area). The TEB is oriented so that the vector followed by the TEB is at a different angle than the vector along which the attached duct lies (arrows). The QuickTime movie of the Z-stack is shown in Additional file 6: Movie S2, and the subregion indicated by the dashed box area is shown in Additional file 6: Movie S3. B. A single Z-slice at a depth of 32 μm reveals the close apposition of SHG-F positive fibers to the TEB caught in an oblique section. C. A 3D reconstruction of the same Z-stack shown in A was made using Volocity software and the transparency chosen to best illustrate the layer of fibers detected by SHG-F (green) in contact with the tip of the TEB. The view looking towards the TEB from the perspective of the coverslip (0o) compared with the view from the opposite direction (180o) demonstrates that the layer of SHG-F-detected fibers (green) lies deeper within the tissue and is most easily visualized from the 180o perspective. D. The image stack used to illustrate Figures 4, 8, and 11 was subjected to 3D reconstruction using Volocity software. A QuickTime animation of the Z-stack is found in Additional file 6: Movie S4. Additional file 6: Movie S5 is a QuickTime animation of the 3D views. In various rotations, the association of layers of fibers with the TEBs is shown, with SHG-B signal predominating at the near surface of the imaged TEB (red) and SHG-F signal predominating at the far surface (green). A-C, Scale bars = 50 μm. [file 1471-2407-13-373-S5.pdf]

# Carmine Alum Whole Mount

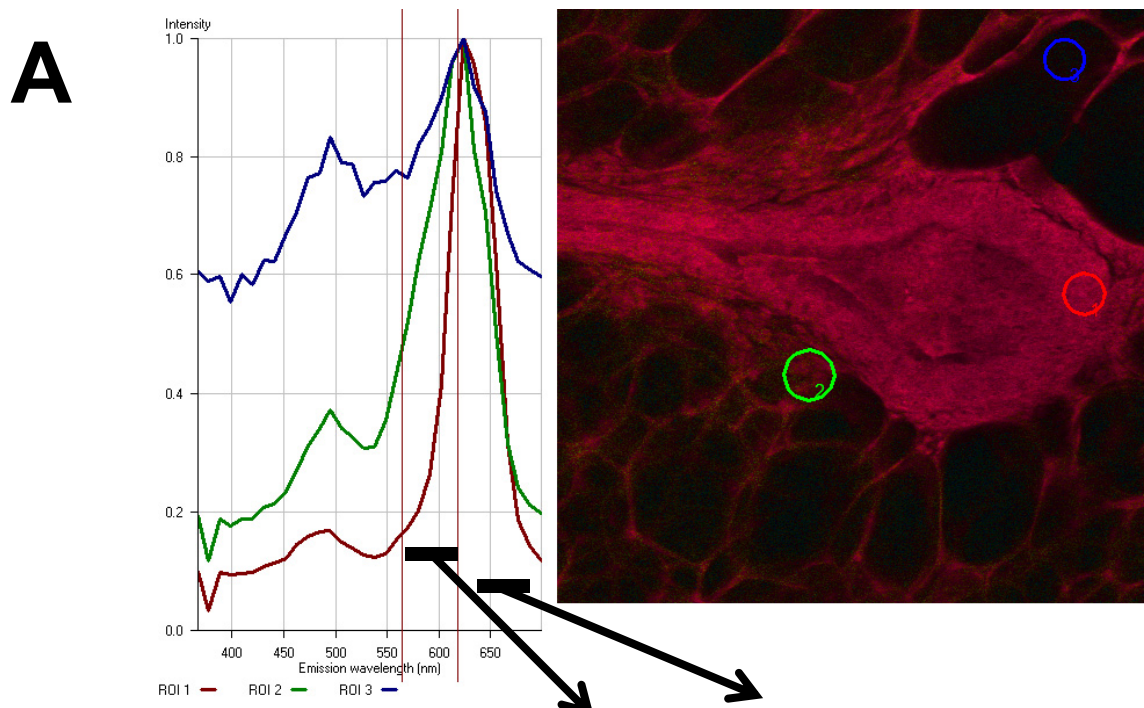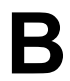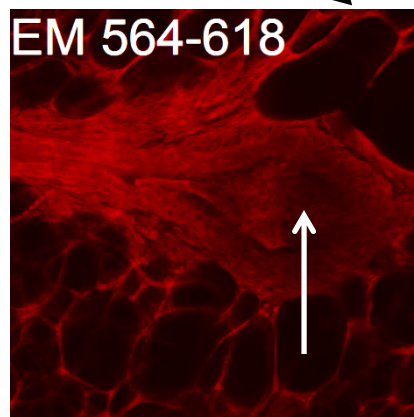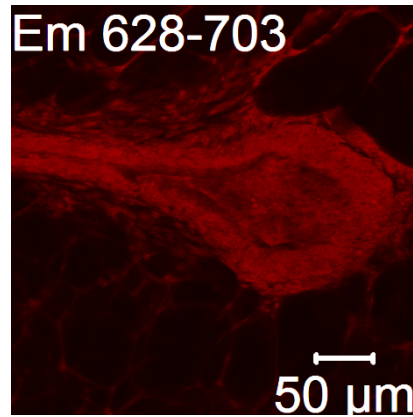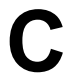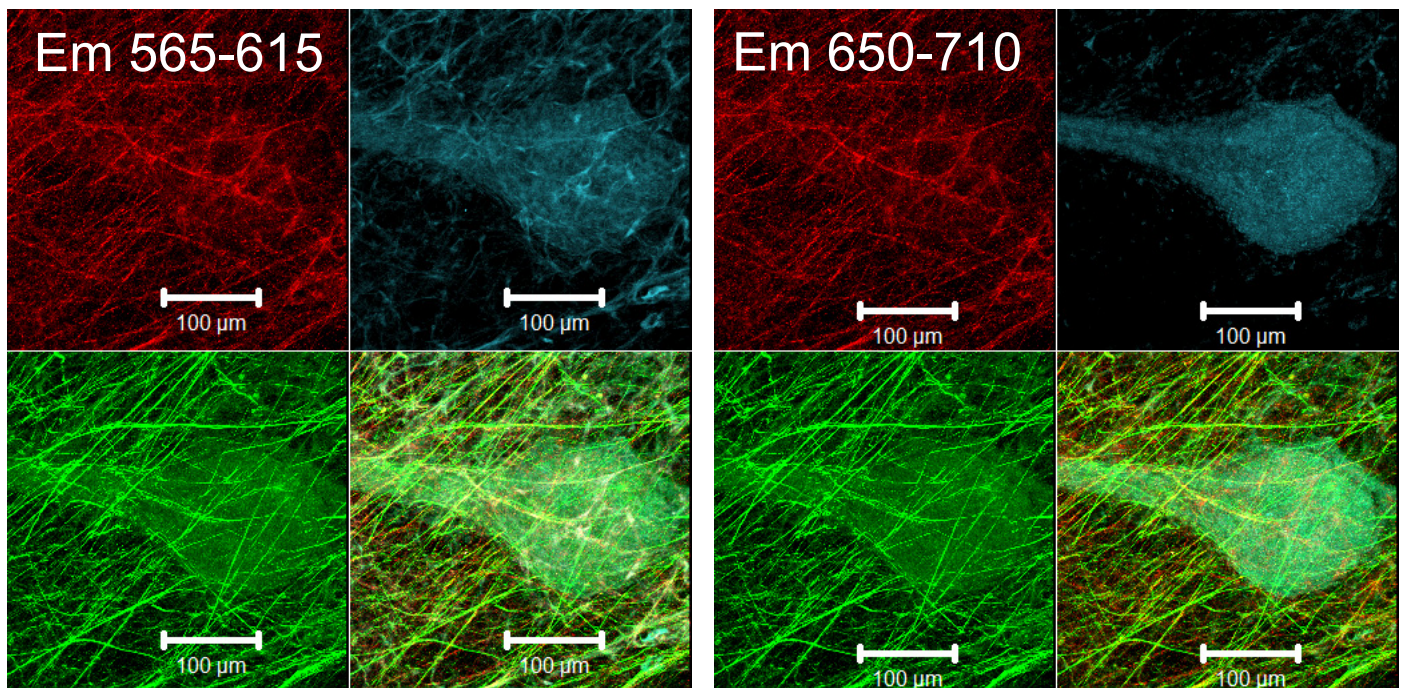

Supplement: Additional file 7: Figure S5. — Effects of emission bandwidth collection on Carmine Alum/ SHG-B/ SHG-F images. A. A lambda scan of a Carmine Alum stained TEB is illustrated together with a graph containing data from three ROIs (blue-background, red-ductal cells near tip of TEB and green-material associated with the TEB margin). B. Images were extracted with defined wavelengths using the Zeiss Meta software. EM 628–703 nm identifies primarily the epithelial tissue whereas the Em 564–618 nm extracted image is striking in the appearance of the TEB-associated material, fat cell outlines, and the shadowing artifact on the TEB (arrow), C. 3D Carmine Alum images (blue) of the same TEB were obtained using Ch3 and fixed emission filters of Em 565–615 (at left) and Em 650–710 (at right) together with SHG-B (red) and SHG-F (green) 3D images. Scale bars = 100 μm. [file 1471-2407-13-373-S7.pdf]

**A**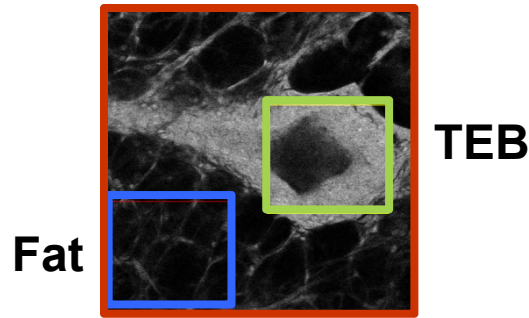**TEB****Fat****B****Carminine Alum**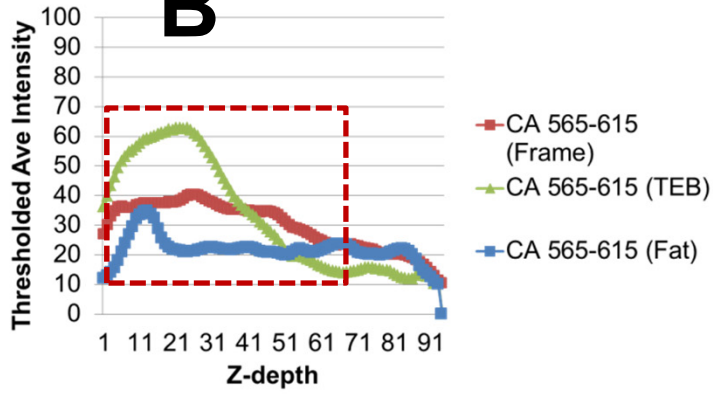**Carminine Alum**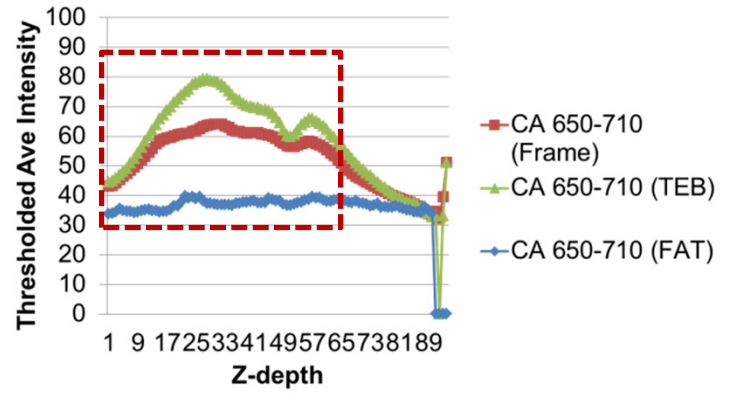**SHG-B**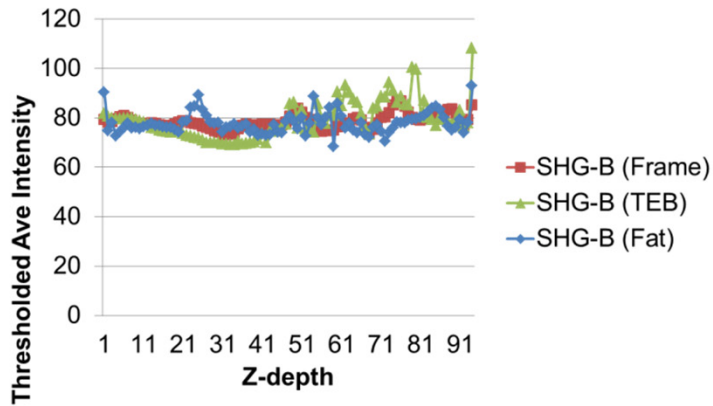**SHG-B**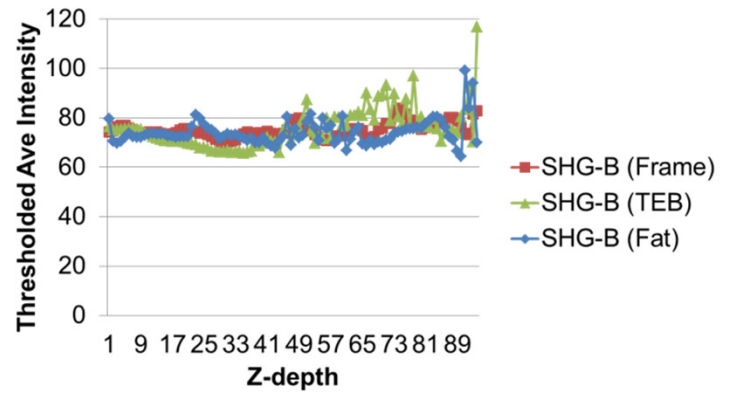**SHG-F**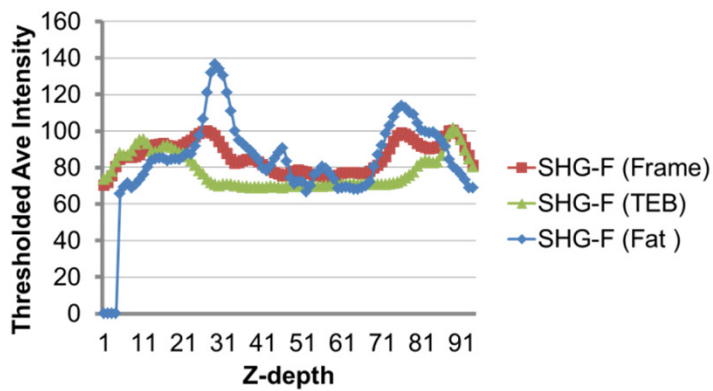**SHG-F**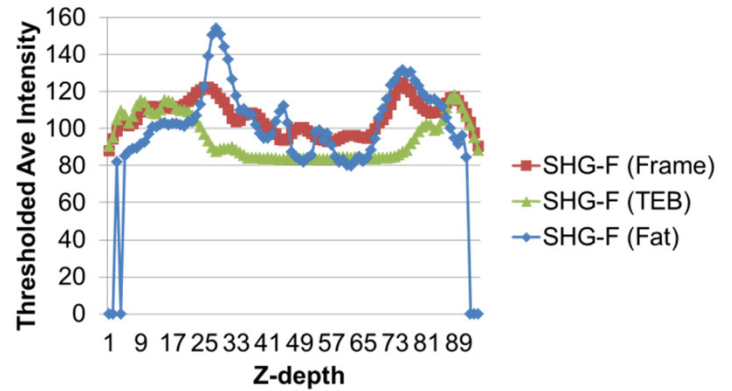

Supplement: Additional file 8: Figure S6 — Quantification of data from Additional file 7: Figure S5C. A-B. To determine the changes in intensity for SHG-B, Carmine Alum, and SHG-F, image stacks were background subtracted and thresholded for Carmine Alum-stained structures, and fibers within the SHG-B and SHG-F images and the average intensity per ROI plotted. ROIs included the entire frame, and the ROIs indicated by the ROI boxes in A over a TEB (TEB) and an area of the stroma containing fat cells (Fat). B. Graphs for each color plane as indicated and Em 565–615 nm (at left) and Em 650–710 nm (at right) are presented. Dashed boxes in the Carmine Alum graphs invite comparison of the loss of stain intensity in the TEB with increasing Z-depth (green trace) in the Em 565–615 nm trace at left relative to the Em 650–710 nm trace at right: improved intensity with increasing Z-depth was observed for Em 650–710 nm. Imaging was identical with the exception of bandpass filter selection. [file 1471-2407-13-373-S8.pdf]

# Shadowing in CA Wholemounds

**A** Smov 3  
Ex 860  
Em 650-710

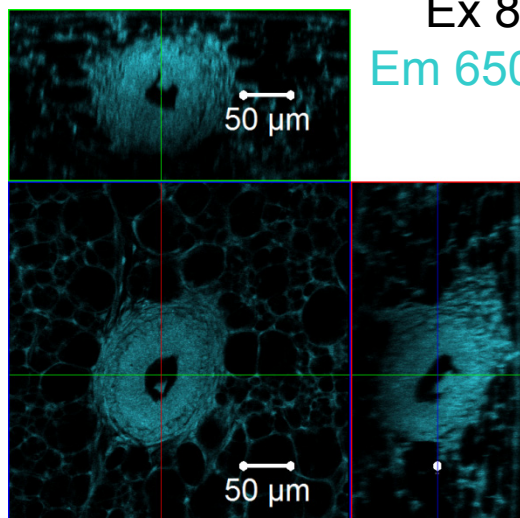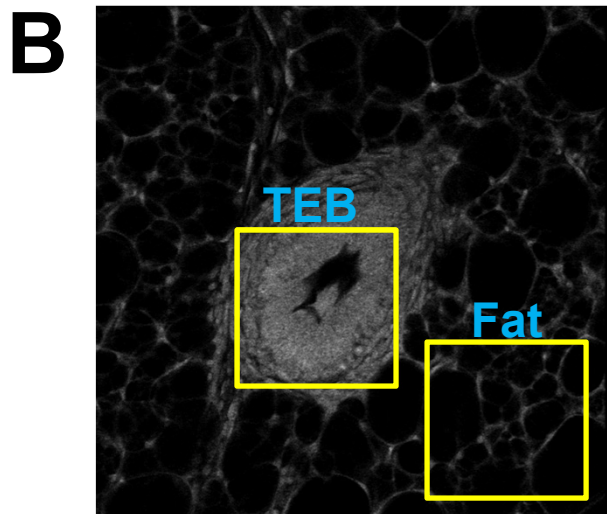

**C**

## Thresholded Area

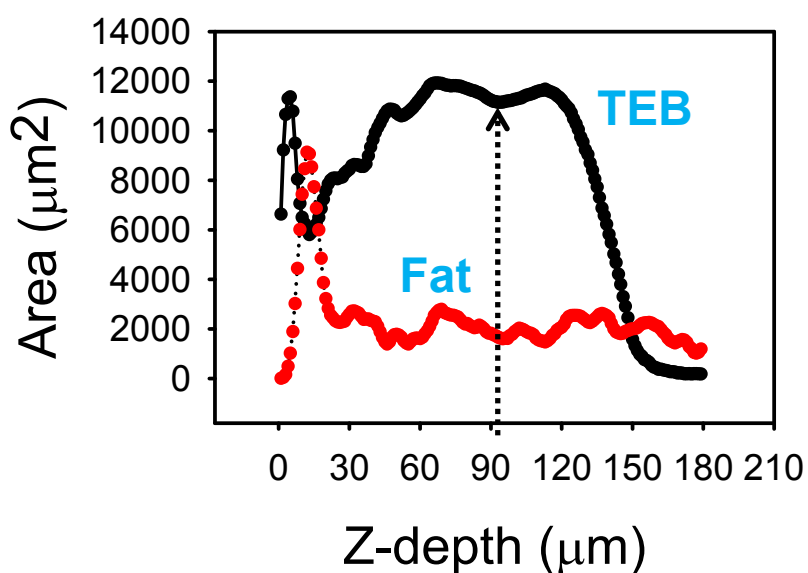

## Average Intensity

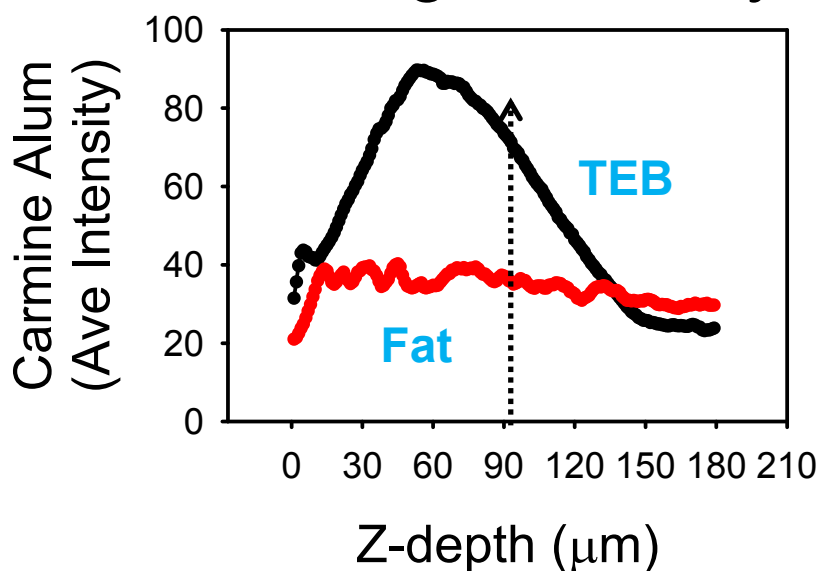

Supplement: Additional file 9: Figure S7 — Quantification of “Shadowing” in Carmine Alum Wholemounts. The TEB presented in Additional file 6: Movie S3 and Additional file 5: Figure S4 A-C was used to quantify the loss of Carmine Alum signal with imaging depth within a TEB. A. Orthogonal views illustrating this Carmine Alum-stained TEB. B. Single plane image illustrating the sites of ROIs for analysis (yellow boxes over TEB and Fat within the stroma). C. Graphs of the data by thresholded area and average intensity. The dotted arrow indicates the midpoint of the TEB where the lumen space contributes to the dip in thresholded intensity. Average intensity drops off beyond the midpoint of the TEB at aprroximtely Z = 90 μm. A, Scale bars = 50 μm. [file 1471-2407-13-373-S9.pdf]

# Unstained Whole Mount

## A

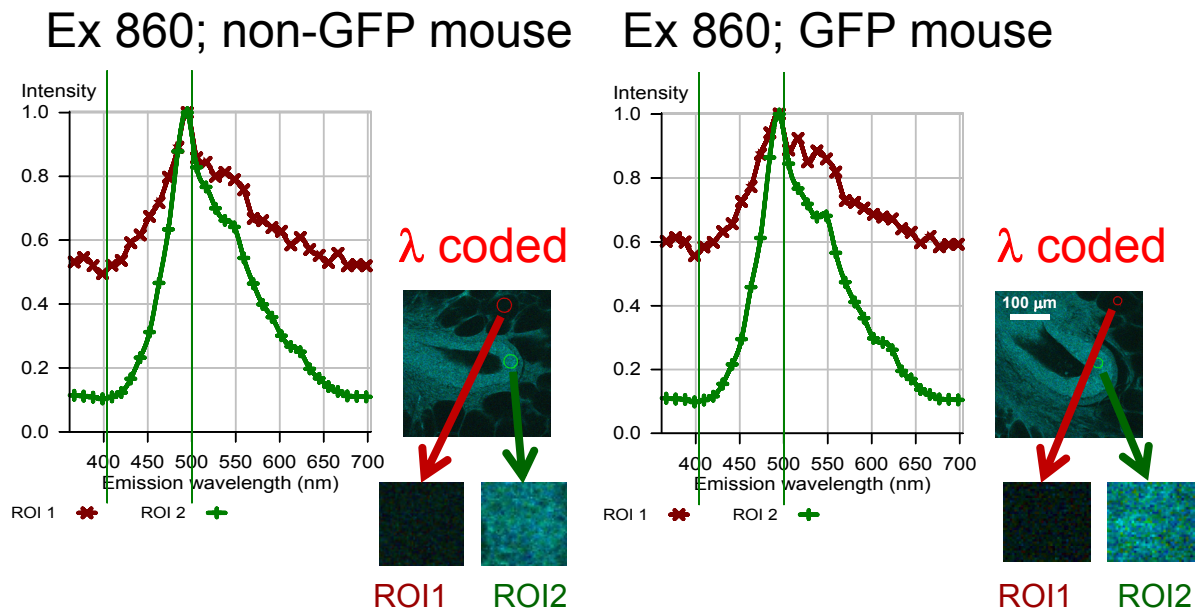

## B

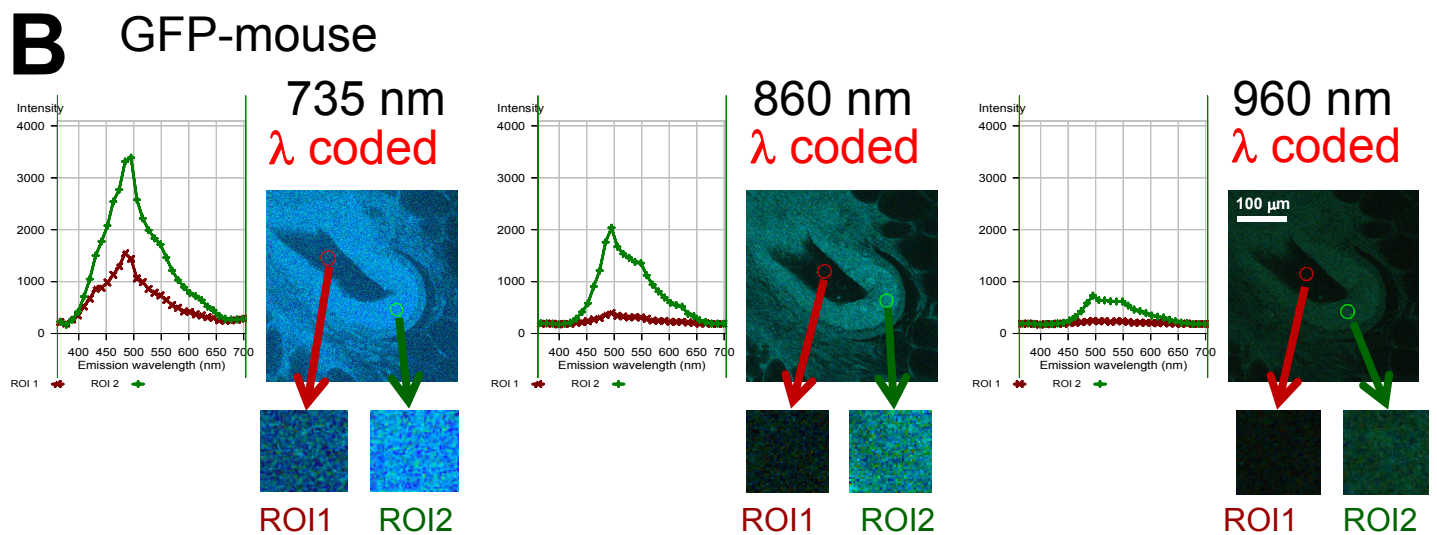

Supplement: Additional file 10: Figure S8 — Comparison of Emission Spectra of GFP and non-GFP Unstained Whole Mount Tissue. A. GFP mice and non-GFP mice were prepared for whole mount without Carmine Alum staining and a lambda scan obtained from Em 361–704 nm using Ex 860 nm. ROI’s in a background area and one within the ductal epithelium were graphed as a function of normalized intensity (arrows indicate insets of ROIs at higher magnification). The intensity scale is normalized for comparison of ROIs. B. GFP mice were prepared for whole mount without Carmine Alum staining and lambda scans were obtained from Em 361–704 nm at Ex 890, 860, and 735 nm. ROI’s in a background area and one within the ductal epithelium were graphed as a function of relative intensity (arrows indicate insets of higher magnification). Average intensity of each ROI is plotted. Scale bars = 100 μm. [file 1471-2407-13-373-S10.pdf]

# Unstained Wholemount Autofluorescence

**A**

EX 860 nm, Lambda scan

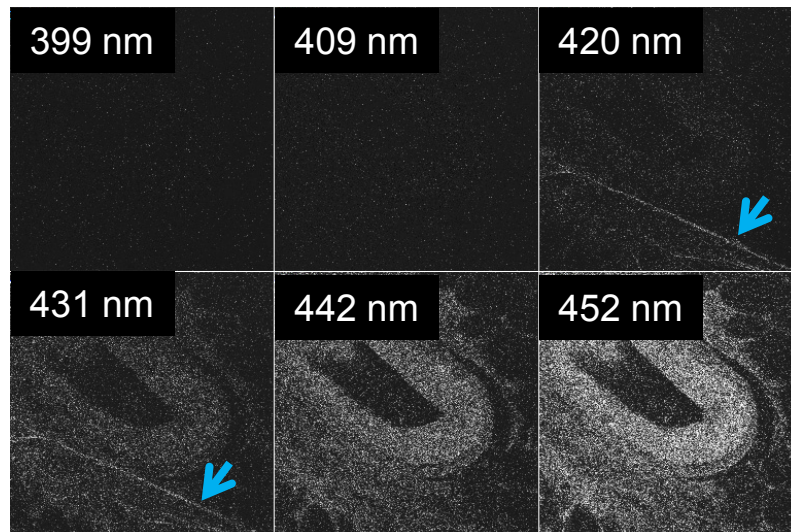

**B**

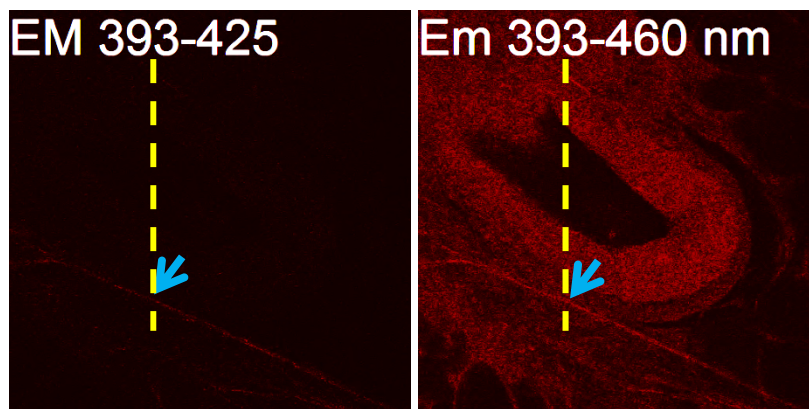

Extract Channels

**C**

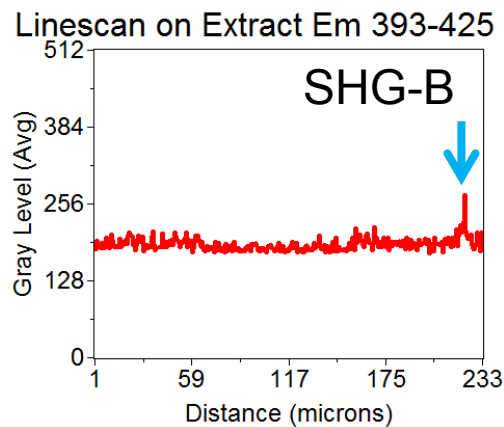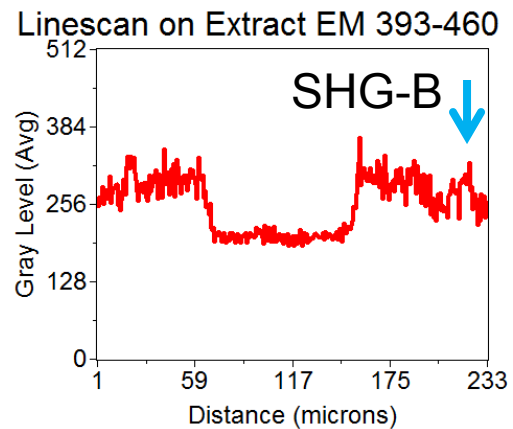

Supplement: Additional file 12: Figure S10 — Reduction in background fluorescence with narrowed SHG-B emission bandwidth. A. A lambda scan was obtained from Em 361–704 nm using Ex 860 nm. A subset of wavelengths is illustrated where the blue arrows indicate the SHG-B signal (420 and 431 nm). B. Bandwidth images were extracted using Zeiss Meta software; blue arrows indicate the SHG-B fibers seen in A. C. Linescans of pixel intensity performed indicated by dashed lines on the two images in B demonstrate the increased signal to noise detection of the fibers (blue arrows). The same TEB was imaged also in Additional file 11: Figure S9A. [file 1471-2407-13-373-S12.pdf]

# Unstained Whole Mount

GFP-mouse

Ex 860 nm

**A**

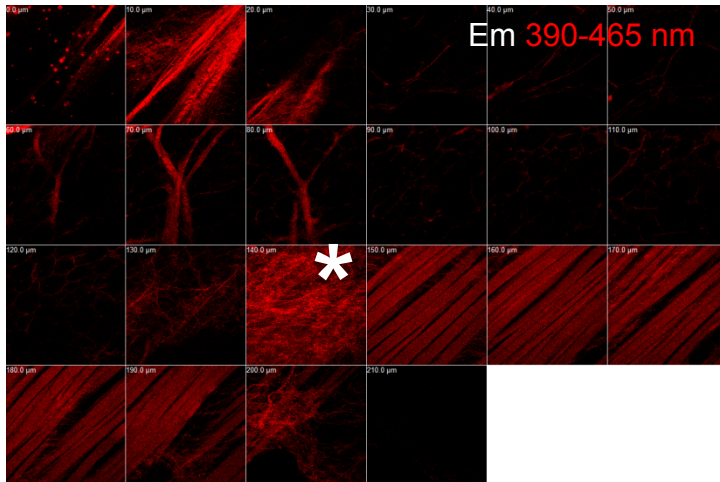

**B**

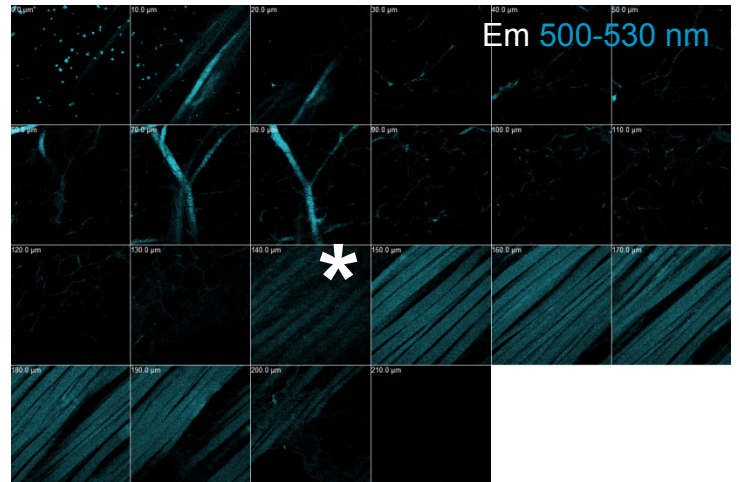

**C**

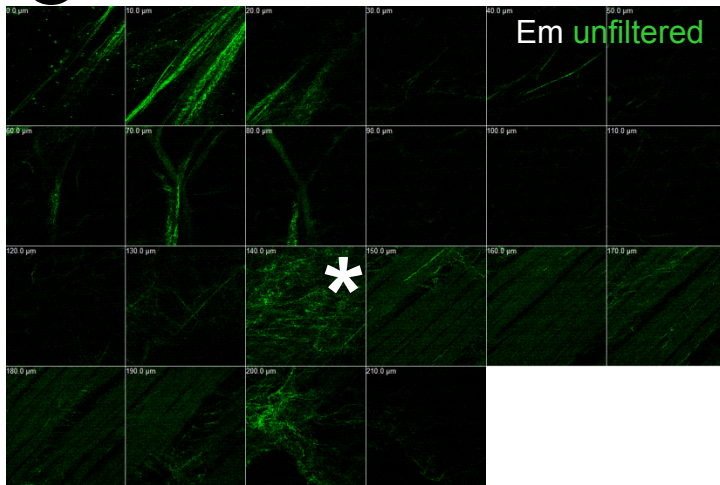

**D**

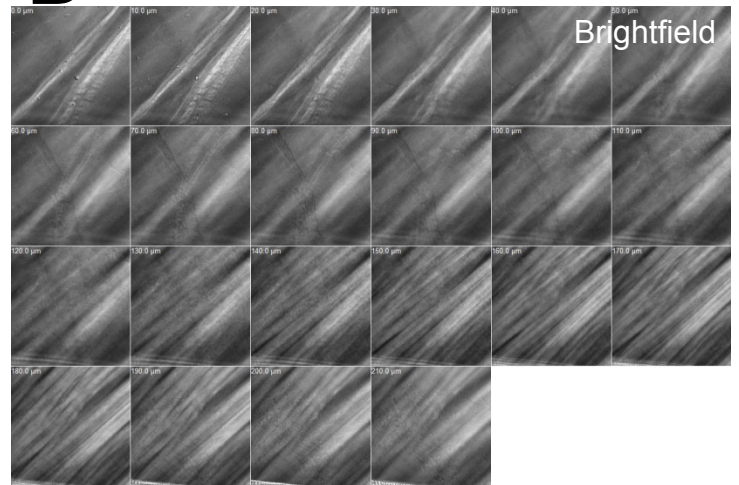

**E**

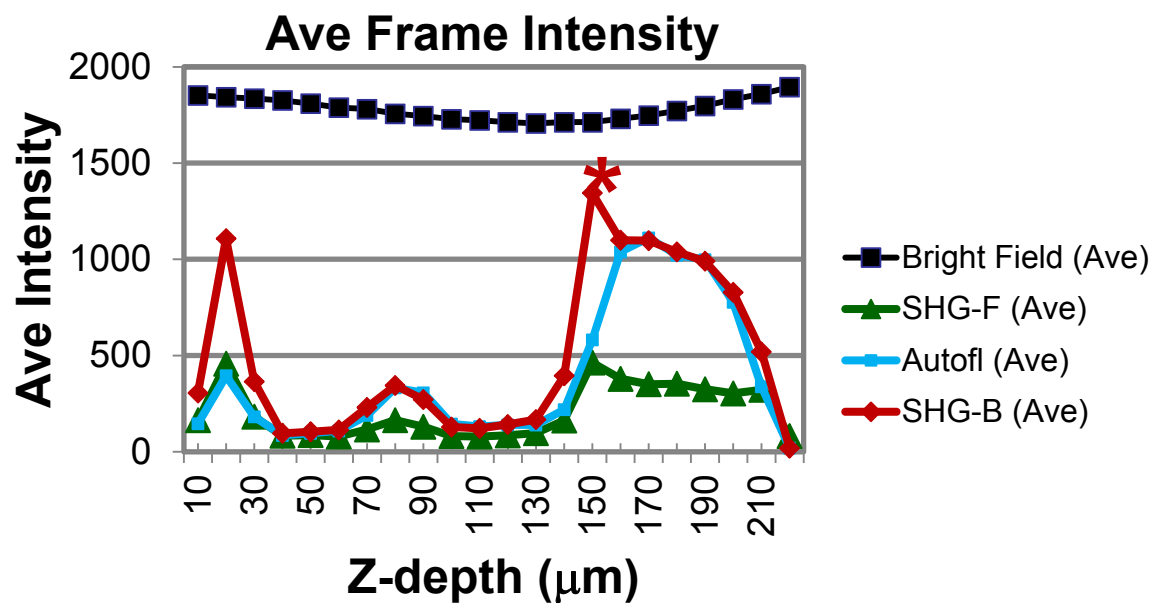

Supplement: Additional file 13: Figure S11 — Imaging unstained whole mounts containing muscle. A-D. A z-stack was obtained for unstained whole mount tissue at Ex 860 with Em 390–465 nm (A, red, SHG-B), Em 500–530 nm (B, blue, autofluorescence), ChD unfiltered (C, green, SHG-F), and brightfield (D). E. The average frame intensity of background subtracted z-stack images is graphed. The asterisk indicates the position of the SHG-B and SHG-F signal observed at 140 μm z-depth in A-C and E. [file 1471-2407-13-373-S13.pdf]
