# Supplementary material for: Three dimensional multiphoton imaging of fresh and whole mount developing mouse mammary glands
Source: BMC Cancer. 2013 Aug 6;13:373. doi: 10.1186/1471-2407-13-373 (PMC3750743; doi:10.1186/1471-2407-13-373)

Unstained Whole Mount

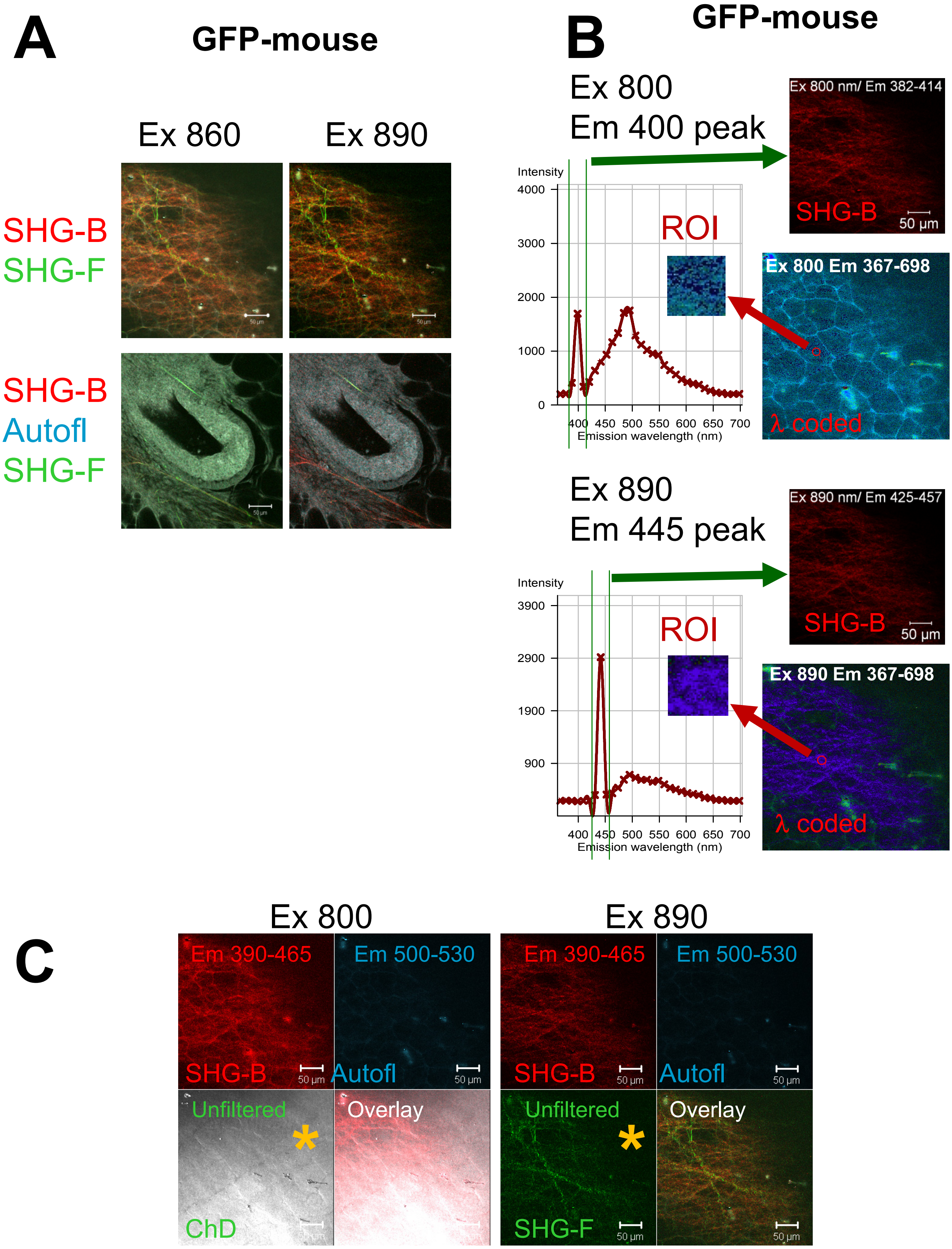

Supplement: Additional file 11: Figure S9 — Comparison of excitation wavelengths for imaging unstained whole mount tissue. A. SHG-B and SHG-F (unfiltered) images were obtained of a single XY plane at a region rich in fibers (upper panels) from a GFP-mouse. At a deeper XY plane that included a median section through a TEB, the autofluorescent background signal was included in a three-color image (lower panels). Comparison of the images at Em 860 and 890 nm reveal little difference in the qualitative information present. B. SHG-B was detected within a focal plane including fibrillar structures and was compared for Ex 735, 800, and 890 nm. None was detected for Ex 735 nm (not shown). An ROI was selected for a region containing fibrils (red circle, red arrow on the lambda coded image) and the average emission intensity plotted for each excitation wavelength. An extracted image was obtained for emission wavelengths containing the SHG maximum intensity: for Ex 800 nm the peak was 400 nm and for Ex 890 nm, the peak was 445 nm (SHG-B, green arrows). C. The transmitted signal was detected for Ex 890 (Ex 890, ChD), but was not detected for Ex 800 or Ex 735 (not shown). A DIC image appears using Ex 800 in ChD, whereas SHG-F appears at Ex 890 (yellow asterisk). Scale bars = 50 μm. [file 1471-2407-13-373-S11.pdf]
